# Functional and structural characterization of mouse Factor H-related B protein unveils a novel dimerization domain shared by FHR-B and FH

**DOI:** 10.3389/fimmu.2025.1522651

**Published:** 2025-01-29

**Authors:** Adrián Martín-Ambrosio Doménech, Silvia González Sanz, Bárbara Márquez Tirado, Lucia Juana-López, Elena Goicoechea de Jorge, Santiago Rodríguez de Córdoba, Héctor Martín Merinero

**Affiliations:** ^1^ Department of Molecular Biomedicine, Center for Biological Research Margarita Salas and Rare Disease Networking Biomedical Research Center, Madrid, Spain; ^2^ Department of Immunology, Faculty of Medicine, Complutense University of Madrid and Research Institute Hospital 12 de Octubre (imas12), Madrid, Spain

**Keywords:** factor H, factor-H related proteins, complement, regulation, complement-related diseases, dimerization

## Abstract

Factor H-related proteins (FHRs) are found in mice, but their equivalence to human FHRs remains uncertain. This study identifies three FHRs in mouse plasma (FHR-B, FHR-C, and FHR-E) and focuses on characterizing FHR-B. Using purified plasma proteins and recombinant mutants, FHR-B was found to form dimers and bind strongly to C3, C3b, iC3b, and C3dg. It also competes with mouse Factor H (mFH) for binding to C3b-coated surfaces and disrupts mFH regulation in hemolysis assays with sheep and guinea pig erythrocytes. These functions are localized to the C-terminal region and are dependent on FHR-B dimerization. Dimerization occurs through the N-terminal region (SCR1-3), which differs from mFH SCR5-7 by only four amino acids and also shares significant homology with human FHR-3 and human FH SCR5-7. In contrast to FHR-1, AUC experiments indicate that FHR-B dimerization is pH-sensitive, reversible and that the monomers in the dimer present the same head to tail orientation. Mutant analyses revealed that mFH SCR5-7 also forms dimers, but less efficiently than FHR-B. Notably, substituting FHR-B Tyr162 (a key residue homologous to the disease-associated Tyr402 in human FH) for His reduces dimerization. We also found that a recombinant FHR-B with a duplicated dimerization domain formed stable dimers but lacked functional activity. Overall, FHR-B shows structural and functional similarities with various human FHRs, suggesting convergent evolution between mouse and human FHRs. Furthermore, this study reveals a novel dimerization domain shared by FHR-B and mouse FH and illustrates the importance of dimerization and monomer orientation in FHRs activity. It also underlines notable differences between human and mice FHRs that should be further explored before modeling FHR-associated human diseases in mice.

## 1 Introduction

**Figure 1:**
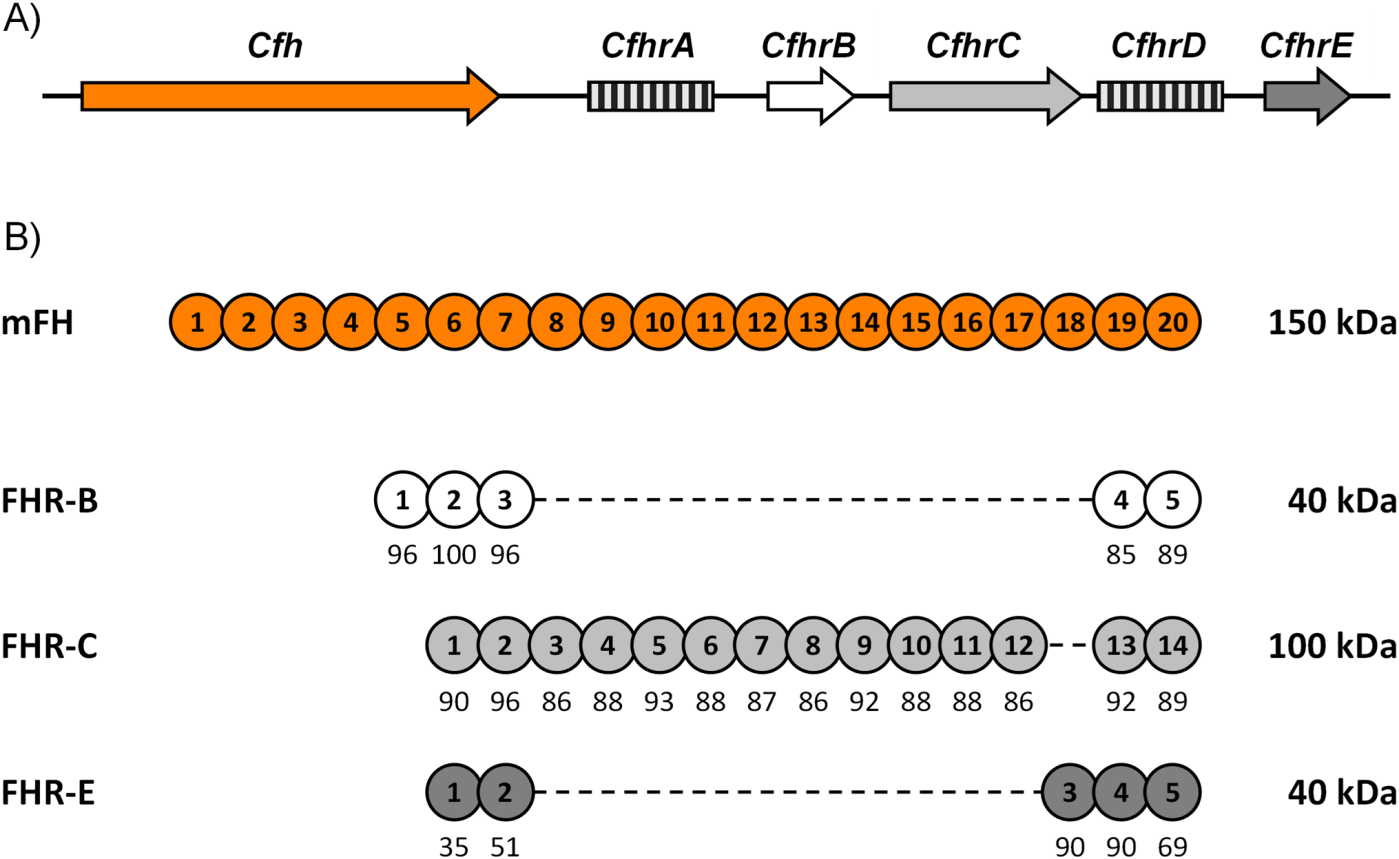
*Cfhrs* genes in mice. **(A)** Genomic organization of the mouse *Cfhrs* genes. Downstream of *Cfh*, there are five *Cfhrs* genes. Genes lacking evidence of transcription are represented by squared boxes. **(B)** Alignment of FHR-B, FHR-C, and FHR-E with mFH. SCRs are numbered inside the circles, and their amino acid sequence similarity to the corresponding mFH SCRs is indicated as percentages.

There are five *Cfhr* genes in mouse chromosome 1 next to the *Cfh* gene within the murine genomic region homologous to the human RCA gene cluster, but evidence that they are functional genes is only available for three of them, *CfhrB*, *CfhrC* and *CfhrE* (1) (**Figure 1A**). At the amino acid level, these murine factor H-related proteins (FHRs) show a much higher sequence identity with mouse factor H (mFH) than with the human FHRs, which makes unclear the equivalence between the mouse and human FHRs. Notably, like their human counterparts, the murine FHRs lack regulatory domains homologous to those found in the N-terminal of mFH, which prevent them from performing as classical complement regulators (**Figure 1B**). Indeed, early experiments using recombinant proteins have described FHR-B as a positive modulator of complement activation on cellular surfaces where it competes with mFH for C3b binding (2–5). These early data have also shown that FHR-B promotes complement activation binding to surface-bound C3b or to other molecules like pentraxin 3 (1), like human FHR-1 and FHR-5 (6, 7).

The activity of the FHRs antagonizing the regulatory role of FH (de-regulation activity) was initially described in humans to explain the association of FHR-1 and FHR-5 mutants with C3-glomerulopathy (C3G) (8, 9) and extrapolated to mutants associated with atypical hemolytic uremic syndrome (aHUS) and to the wild-type FHRs (10). Later on, *in vitro* experiments with FHR-1 and FHR-4 showed that the FH de-regulation activity of the FHRs may involve two non-exclusive mechanisms: competition with FH for binding to surface-bound C3b and promotion of complement activation by sustaining the formation of alternative pathway (AP) C3 convertases at certain surfaces (2, 11). Recent functional and structural data using FHR-1 and disease-associated FHR-1 mutants have advanced our knowledge of the physiological role of FHR-1 and helped us to delineate a novel mechanistic framework for the association of FHR-1 with various diseases like aHUS, C3G, IgA-nephropathy (IgAN) and age-related macular degeneration (AMD) (12–14). According to these ideas, acquisition of the capacity to bind sialic acids by the aHUS-associated FHR-1 mutants generate a potent FH competitor that reduces the protection of endothelial surfaces from complement-mediated damage. On the other hand, in conditions such as C3G, IgAN and AMD, which encompass various stages, one of which is the generation of a C3 opsonized surface, our proposal is that an excessive functional FHR-1/FH activity ratio, caused by gain-of-function FHR-1 mutants (like those duplicating the N-terminal dimerization domain) or elevated FHR-1 levels, will exacerbate the activation of complement at those opsonized surfaces in a pathological way.

The studies performed here were designed to extensively characterize the mouse FHR-B to better define its functional homologies and differences with the human FHRs. We report that FHR-B is a dimeric protein resembling human FHR-1, FHR-2 and FHR-5, but involving a different dimerization domain with distinct structural and functional characteristics. Our studies have also unveiled that this novel FHR-B dimerization domain is conserved in mFH and perhaps also in the human FHR-3 and human FH. We expect that the knowledge provided here will advance our knowledge of the FH and FHRs disease associations and paved the way for modeling the FHR-associated human diseases in mice.

## 2 Materials and methods

### 2.1 Purification of FHR proteins from mouse plasma and proteomic analysis

mFH was purified to homogeneity from mouse ethylenediaminetetraacetic acid (EDTA)-plasma by affinity chromatography using the anti mFH monoclonal antibody (mAb) 2A5 (a gift of Prof, P.Morgan, Cardiff University) and gel filtration chromatography. We generated a rabbit polyclonal antibody (pAb) raised against this purified mFH (**Supplementary Figure 1**) and the protein G-purified IgG from this antiserum was coupled to a Sepharose column that then, was used to affinity-purify mFH-crossreacting proteins from the EDTA-plasma of FH deficient mice (*Cfh^-/-^*). The eluted proteins were trypsin digested and a nano-scale liquid chromatographic tandem mass spectrometry (nLC-MS/MS) analysis was performed following established protocols at the Proteomics and Genomics Facility of the CIB-CSIC, a member of ProteoRed-ISCIII network (15, 16). As a negative control, similar amount of *Cfh^-/-^* mice EDTA-plasma was loaded into a Sepharose column with the protein G-purified IgG from a non-immunized rabbit control. The eluted proteins from this control experiment were trypsin digested and analyzed as described above.

### 2.2 Generation, expression and purification of mouse FHRs recombinant proteins

The cDNAs of human FHR-1 (UniProt Q03591) and mFH (UniProt P06909) in the pEZ M02 vector were obtained from GeneCopoeia, while that of mouse FHR-B (UniProt Q4LDF6) in the pcDNA3.1+/C (K) DYK vector was purchased from GenEZ ORF (Genscript). The two C-terminal SCR domains of mouse FHR-B, FHR-C (UniPort E9Q8B5) and FHR-E (UniProt Q61406) were generated with GeneArt Gene Synthesis (Thermo Scientific) in the pMA-T vector flanked by the *AgeI* (5’) and *MluI* (3’) restriction sites and cloned into the pMA-T vector.

For the generation of the FHR-1_1-3_::mFH_19-20_ hybrid protein, a new *AgeI* restriction site, which resulted in synonymous codons, was generated between SCRs 3-4 of FHR-1 and SCRs 18-19 of mFH by site-directed mutagenesis (New England Biolabs, #E0554S). FHR-1_1-3_ fragment was extracted by digesting the pEZ-M02 vector containing FHR-1 with *SacI* (uniquely present upstream of the ORF) and *AgeI* (previously generated), and it was cloned into the pEZ-M02 vector containing the mFH from which the mFH_1-17_ fragment was previously stripped by digestion with *SacI* and *AgeI*. The final construction was cloned into an *in-house* pCMV promoter expression vector flanked by *NheI* (5’) and *MluI* (3’).

To generate the hybrids FHR-1_1-3_::FHR-B_4-5_, FHR-1_1-3_::FHR-C_13-14_, FHR-1_1-3_::FHR-E_4-5_ and FHR-1_1-3_::FHR-5_8-9_ the fragments FHR-B_4-5_, FHR-C_13-14_ and FHR-E_4-5_ were extracted from the pMA-T vectors by digesting with *AgeI* and *MluI*, and the fragment FHR-5_8-9_ was amplified, incorporating restriction sites *AgeI* (5’) and *MluI* (3’), from a pCI-Neo vector containing FHR-5 (UniProt Q9BXR6) cDNA. Fragments extracted were cloned into the *in-house* pCMV promoter expression vector containing the hybrid FHR-1_1-3_::mFH_19-20_ from which the mFH_19-20_ fragment was previously stripped by digestion with *AgeI* and *MluI*. We also generated a monomeric variant of FHR-1_1-3_::FHR B_4-5_ hybrid protein by introducing mutations to the key residues of the dimerization domain in FHR 1 as described (*FHR-1_1-3_::FHR-B_4-5_) (8).

The hybrid FHR-1_1-2_::FHR-B_1-3_ was generated by In-Fusion cloning technology (Takara Bio, #639648) as indicated by the manufacturer. A variant of the native FHR B (rFHR-B) was created by introducing a His-tag sequence between the signal peptide and the SCR1 of the protein, followed by a 3C protease cleavage site to eliminate the His-tag after purification.

The N-terminal mutants FHR-B_Y162H_ and FHR-B_T161A,I166V_ where generated by site directed mutagenesis (New England Biolabs, #E0554S) on the rFHR-B. Depletion of SCR1 (FHR-B_Del.1_) was performed by amplifying the His-tagged full-length FHR-B using opposed primers binding to the 3C protease cleavage site and to the start of SCR2 and circularizing the PCR product. To duplicate SCRs 1-3 (FHR B_Dup.1-3_) we linearized the His-tagged full-length FHR-B by digesting it with *MluI* between SCRs 3-4 and ligating it with the fragment comprising FHR-B SCRs 1-3 amplified in parallel.

All constructs were transferred to an *in-house* pCMV promoter expression vector, their sequences confirmed by Sanger sequencing and transfected for protein expression into mammalian FreeStyleTM 293-F cells using the FreeStyleTM 293 Expression System (Invitrogen, #K900001). The protein expression and concentration in the supernatant was assessed by western-blot and enzyme linked immunosorbent assay (ELISA). The hybrid proteins containing FHR-1_1-3_ were purified by affinity chromatography using the monoclonal antibody anti-hFHR-1 2C6 (17) coupled to a CNBr-activated sepharose 4B (GE Healthcare, # 17043001) column. The His-tagged wild type and mutant rFHR-B proteins were purified using the HisTrap HP columns (GE Healthcare, #17524701) using 45mM of imidazole (Merck, #4015-100GM) in the supernatant and washing buffer and eluting with 250mM of imidazole. The proteins were dialyzed against PBS before overnight digestion at 4°C with His-tagged 3C protease (produced by Protein Chemistry Facility in CIB-CSIC) and was then charged again into the HisTrap HP where the untagged protein was separated from the protease in the flow-through. All proteins were dialyzed against PBS, aliquoted and stored at -80°C.

### 2.3 Generation of specific antibodies to mouse FHR-B and measurement of FHR-B levels in mice plasma

A rabbit polyclonal antibody was raised against the purified untagged rFHR-B protein using standard protocols and the IgG from this antiserum purified using protein-G coupled to a Sepharose column. Cross-reactivities with mFH were removed from the anti-mouse FHR-B rabbit IgG using mFH coupled to a Sepharose column. The mFH-adsorbed rabbit IgG anti-mouse FHR-B shows no cross-reactivity with mFH and was used to determine the concentration of FHR-B in the plasma of male and female C57BL/6, C3 deficient (*C3^-/-^*) and *Cfh^-/-^* mice using a sandwich ELISA as follows. Clear flat-bottom polystyrene 96-well microplates (Corning, #3590) were coated overnight at 4°C with *in-house* rabbit polyclonal anti-FHR-B adsorbed against mFH at 1 mg/ml in PBS. Plates were washed three times in wash buffer (Tris 50 mM, pH 7.4, NaCl 150 mM and 0.2% Tween 20) before blocking with 100 µl per well with blocking buffer (wash buffer plus 1% BSA) for 1h at room temperature (RT). After additional washes the appropriate dilution (1:1700 and 1:3400) of the tested mice serum in blocking buffer was added and incubated for 1h at RT. Then, the plates were washed before addition of 100µL of the *in-house* rabbit polyclonal anti-FHR-B adsorbed against mFH and conjugated with biotin diluted in blocking buffer for 1h at RT. The plates were then washed and incubated with Streptavidin conjugated with horseradish peroxidase (HRP) (DAKO, #P0447) for 30 min at RT. Finally, plates were washed before developing with 100 µl of 3,3´, 5,5´-tetramethylbenzidine (TMB) solution (Kementec, #5320A) for 3 min before stopping the reaction with 100 µl of sulfuric acid 0.1M. Optical density (OD) values were determined at wavelength of 450 nm.

### 2.4 Hemolytic assays

Complement de-regulation activities were performed using the guinea pig (GP) or the sheep (Sh) hemolysis assay (18). GP erythrocytes (GP-E) (TCS Biosciences, #PB029AP) or Sh erythrocytes (Sh-E) (Durviz, #RS0001) were washed several times with complement fixation diluent (CFD) buffer (2.5 mM barbital, 1.5 mM sodium barbital, 144 mM NaCl and 10 mM EGTA, pH 7.2–7.4) supplemented with 7mM MgCl_2_ (AP-CFD) before adjusting the concentration to 3x10^8^ cells/mL. 50 µl of erythrocytes were incubated with 15% C57BL/6 mouse serum and incubated for 1h in a bath at 37°C with or without appropriate dilutions of the tested proteins in a final volume of 100 µl. Blanks were prepared in CFD buffer containing 20mM EDTA (CFD-EDTA) adding erythrocytes and 15% C57BL/6 serum. After incubation, the reaction was stopped by the addition of 100 µl of CFD-EDTA buffer. The mixtures were then centrifuged at 3000rpm at 4°C for 5 min and the absorbance of the supernatant determined at 414 nm.

### 2.5 C3b binding experiments

Clear flat-bottom polystyrene 96-well microplates (Corning, #3590) were coated overnight at 4°C with 50 µl of mouse C3b (or human C3b) at a concentration of 5 µg/ml in PBS buffer. Plates were washed three times in wash buffer (Tris 50 mM, pH 7.4, NaCl 150 mM and 0.2% Tween 20) before blocking with 100 µl per well with blocking buffer (wash buffer plus 1% BSA) for 1h at RT. After additional washes the appropriate dilution of the tested proteins in blocking buffer were added and incubated for 1h at RT. Then, the plates were washed before addition of 1 µg/mL mAb anti-hFHR-1 2C6 in 100 µl blocking buffer for 1h at RT. The plates were then washed and incubated with a goat anti-mouse IgG conjugated with HRP (DAKO, #P0447) for 30 min at RT. Finally, plates were washed before developing with 100 µl TMB solution (Kementec, #5320A) for 3 min before stopping the reaction with 100 µl of sulfuric acid 0.1M. OD values were determined at wavelength of 450 nm.

### 2.6 Analytical ultracentrifugation

The sedimentation velocity of different proteins was determined through analytical ultracentrifugation (AUC) as described (19). Samples of protein in PBS were loaded (320 µl) into 12 mm epon-charcoal standard double-sector centerpieces (Beckman-Coulter Inc.) and the assays were performed at 48000 rpm in a XL-I analytical ultracentrifuge (Beckman-Coulter Inc.) equipped with both UV-VIS absorbance and Raleigh interference detection systems, using an An-50Ti rotor. Sedimentation profiles were recorded simultaneously by Raleigh interference and absorbance at 230 and 260 nm. Differential sedimentation coefficient distributions were calculated by least-squares boundary modeling of sedimentation velocity data using the continuous distribution c(s) Lamm equation model as implemented by SEDFIT (20). All AUC experiments were carried out in the Molecular Interactions Facility (CIB-CSIC), AENOR code ER-0286/2009.

### 2.7 Immunofluorescence assays

Mouse kidneys were extracted and washed with PBS. The organs were embedded in Tissue-Tek^®^ optimal cutting temperature (OCT) Compound (Sakura, #4583) and the blocks were instantly frozen in an isopentane bath chilled with dry ice. The blocks were stored at -80°C until used. Cryostat sections (5µm) were cut over Superfrost Plus microscope slides (Thermo Scientific, #J1800AMNZ) at -18°C in a Leica CM 1800 cryostat. The slides were kept at -20°C for less than 3h before initiating the immunofluorescence protocol. Then, the slides were tempered and washed in PBS for 5 min at RT and fixated with cold acetone (-20°C) for 10 min. The tissue sections were surrounded with the Immedge hydrophobic pen (Vector Laboratories, #NC9545623) in order to reduce sample volumes before washing again with PBS. For the protein binding experiments, 50 µl of the appropriate dilutions of the tested proteins in PBS 1% BSA were added and incubated for 40 min at RT. After washing with PBS, tissue samples were blocked with PBS containing 3% BSA for 20 min at RT and with the Biotin Blocking System (DAKO, #X0590). Bound proteins were detected with appropriated dilution of the corresponding biotin-conjugated antibodies followed by either streptavidin labeled with Alexa Fluor 488 (M. Probes, #S 32354) 1/1000 or horse anti-rabbit IgG-DyLight488 (Vector, #DI-1088-1.5) 1/4000, diluted in PBS containing 1% BSA and incubated for 40 min at RT. To remove sialic acids, the tissue sections were incubated with 50µl of *Clostridium perfringens* neuraminidase (Sigma Aldrich, #N2876-25UN) at 10U/ml diluted in PBS for 90 min at 37°C. Endogenous FHR-B was detected with mFH-adsorbed rabbit IgG anti-mouse FHR-B followed by a horse anti rabbit IgG-DyLight488 (Vector, #DI-1088-1.5). C3 deposits were detected with an anti-mouse C3 conjugated with fluorescein isotiocyanate (FITC) (MP Biomedical, #55557). All tissue sections were mounted with *in-house* prepared Mowiol and stored overnight in the dark at 4°C.

### 2.8 Competition between FHR-B and mFH for binding to opsonized sheep erythrocytes

Sh-E in CFD-EDTA were sensitized using a 1:1600 dilution of rabbit anti-ShE antiserum (Siemens Amboceptor, #ORLC25), incubated for 30 minutes at 37°C and then washed twice with CFD-EDTA buffer. Sensitized Sh-E, at an OD of 0.5 at 414nm in CFD, were then opsonized with a 1:20 dilution of normal human serum depleted of FH, FHR-1, FHR-2, FHR-5 and FB (NHSΔFHRs&FB) and 20 mg/ml of OmCI, an inhibitor of C5 activation, for 30 minutes at 37°C. As a negative control, opsonization was also performed in the presence of CFD-EDTA. The reactions were stopped with 2 ml of CFD-EDTA and cells were washed twice in PBS. Cell opsonization was confirmed by flow cytometry experiments with an *in-house* monoclonal mouse anti-human C3 antibody (12.17) and a phycoerythrin-labeled goat anti-mouse IgG secondary antibody (eBiosciences, #12-4010-82). The binding of mFH to either opsonized or non-opsonized Sh-E was then titrated. To do so, increasing amounts (3–100 nM) of mFH in-house labeled with Alexa Fluor 488 (mFH^AF488^) (Invitrogen, # P30012) were incubated with the cells, for 1 hour at 4°C in PBS containing 0.1% BSA. mFH^AF488^ binding was then analyzed by flow cytometry. For the mFH binding competition assays, 50 nM mFH^AF488^ and increasing amounts (25, 50, 100 or 200 nM) of the competitors (mFH, FHR-1 and FHR-B) were incubated for 1 hour at 4°C with the opsonized Sh-E. As a negative control for the competition 25, 50, 100, or 200 nM of BSA was also incubated with mFH^AF488^. A molecule was considered a competitor of the FH binding to opsonized Sh-E when the mFH^AF488^ geometric mean determined by flow cytometry in the presence of that molecule was significantly reduced, as compared with the geometric mean obtained for the negative control.

### 2.9 Activation of human complement by cryostat kidney sections from *Cfh^-/-^*;*Cfhr^-/-^* mice

Cryostat sections from FH and FHRs deficient (*Cfh^-/-^*;*Cfhr^-/-^*) mice kidneys were washed in PBS containing 1% BSA and then incubated for 30min at 37°C with 10% of human ethylene glycol-bis(β-aminoethyl ether)-N,N,N′,N′-tetraacetic acid (EGTA)-serum that has been depleted of FHR-1, FHR-2 and FHR-5 (NHSΔFHRs) proteins and with sufficient amount of FH added to just prevent any complement activation upon incubation with cryostat sections from C57BL/6 mice kidneys. Parallel experiments were run with or without rFHR-B added to the human EGTA-serum. After few washes in PBS, sections were processed to block endogenous biotin using the biotin blocking system (DAKO, #X0590), following the instructions by the manufacturer. Biotinylated mAb 12.17 was added at 5μg/mL in PBS containing 1% BSA and incubated at RT for 40min in the humid chamber. After extensive washes, the sections were finally incubated with streptavidin labeled with Alexa Fluor 488 (M. Probes, #S 32354) diluted 1:1000 in PBS containing 1% BSA for 30min at RT. After additional washes the sections were incubated with 4′,6-diamidino-2-fenilindol (DAPI) (Sigma Aldrich, #D9542-5MG) for 2min and mounted with *in-house* Mowiol. Image analysis was performed with software ImageJ.

### 2.10 FI-cofactor activity

FI-mediated cofactor activity of FHR-B was tested in the fluid phase by incubating hC3b (270 nM) and hFI (40 nM) with increasing amounts of FHR-B (170 nM–5400 nM) in PBS at 37°C for 45 minutes. Background activity was assessed in the absence of FHR-B. FHR-B potentiation of FI-mediated cofactor activity of FH was tested in the fluid phase by incubating hC3b (270 nM), hFI (40 nM), and hFH (3.5 nM) with increasing amounts of FHR-B (85 nM–1360 nM) in PBS at 37°C for 45 minutes. Background FI-mediated cofactor activity of FH was assessed in the absence of FHR-B. To analyze the impact of FHR-B N-terminal mutants (FHR-B_T161A,I166V_, FHR-B_Y162H_, FHR-B_Dup.1−3_, FHR-B_Del.1_), FHR-1_1-3_::FHR−B_4-5_ and *FHR-1_1-3_::FHR−B_4-5_ on the FI-cofactor activity of FH, we incubated hC3b (270 nM), hFI (40 nM), and hFH (0.8 nM) with 1500 nM of each FHR-B mutant at 37°C for 45 minutes. FHR-B (1500 nM) was used as a reference. The results of the incubations were analyzed by loading the products onto an sodium dodecyl sulfate polyacrylamide gel electrophoresis (SDS-PAGE) and staining with Coomassie Brilliant Blue R-250 (Bio-Rad, #161-0400). The bands corresponding to the α’ and β chains of C3b, as well as the α40 chain (when cleavage occurred), were measured by densitometry using the Image Lab™ program associated with the ChemiDoc™ (Bio-Rad) imaging system.

### 2.11 Animals

C57Bl/6J and C3 knockout mice (*C3^−/−^*) on C57Bl/6J background were purchased from The Jackson Laboratory and maintained in the animal facilities from Centro de Investigaciones Biológicas-CSIC. *Cfh* knockout mice (*Cfh^−/−^*) and kidneys from *Cfh-Cfhr*s double knockout mice (*Cfh^−/−^; Cfhrs^−/−^*) were a gift from Prof. Matthew Pickering at Imperial College London.

### 2.12 Statistical analysis

All experiments were performed in triplicate and conducted in at least three independent assays. All figures present data from a representative assay, displaying the mean and standard deviation of the triplicates. Statistical analyses, when applicable, were performed using GraphPad software, version 8. Comparisons of functional activities were made using Student’s t-test for independent samples against the reference protein. A p-value of <0.05 was considered statistically significant.

### 2.13 Ethics

Ethics approval for this research (S2022/BMD-7278) and animal work was approved by our institute ethics committee and the animal experimental committee of the Spanish Ministry of Agriculture (PROEX 005/19 and 138.3/24).

## 3 Results

### 3.1 Detection of FHR-B, FHR-C and FHR-E in mouse plasma

**Figure 2:**
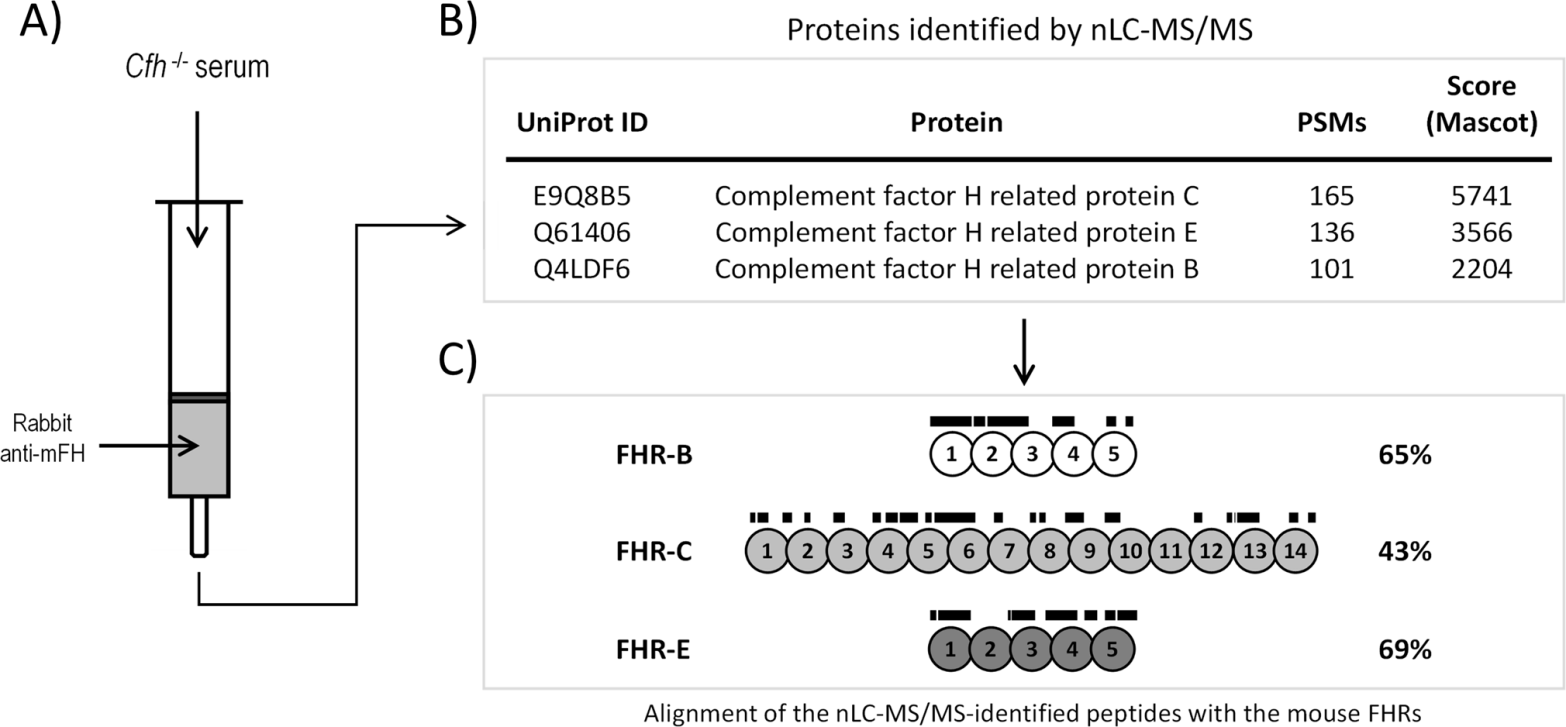
Identification of FHR-B, FHR-C and FHR-E in mice plasma using a proteomic approach. **(A)** Mouse FHR proteins were purified from C57BL/6 *Cfh^-/-^* plasma by affinity chromatography using protein G-purified rabbit IgG polyclonal antibody anti-mFH (see Material and Methods). **(B)** Eluted proteins were digested and analyzed by nLC-MS/MS, which identified several peptides unique to mouse FHR-B, FHR-C, and FHR-E proteins (See **Supplementary Materials** for a list of these peptides and additional proteins identified in these analysis). No peptides corresponding to FHR-A nor FHR-D were found. Importantly, no peptides unique to mouse FHRs proteins were identified in the analysis using a control Sepharose column coupled to a non-immune rabbit IgG. **(C)** Lines above SCRs identify the position of the unique peptides to mouse FHR-B, FHR-C, and FHR-E proteins, to illustrate that they provide an almost complete coverage of these proteins.

We leverage the high sequence similarity between mouse FHR proteins and mouse FH to affinity-purify FHR proteins from the plasma of *Cfh^-/-^* mice. This was done by coupling to a Sepharose column the protein G-purified IgG from a rabbit immunized against purified mFH (**Figure 2A**) (See Materials and Methods). The eluted proteins were analyzed by nLC-MS/MS, which identified several peptides unique to mouse FHR-B, FHR-C, and FHR-E proteins (**Figure 2B**; **Supplementary Table 1**). These results confirmed the presence of these proteins in plasma (**Figure 2C**), as previously suggested (1), but did not detect FHR-A and FHR-D. Importantly, no mouse FHRs proteins were retained by a control Sepharose column coupled to a non-immune rabbit IgG (**Supplementary Table 2**).

### 3.2 Structural and functional similarity between mouse FHR-B and human FHR-1

**Figure 3:**
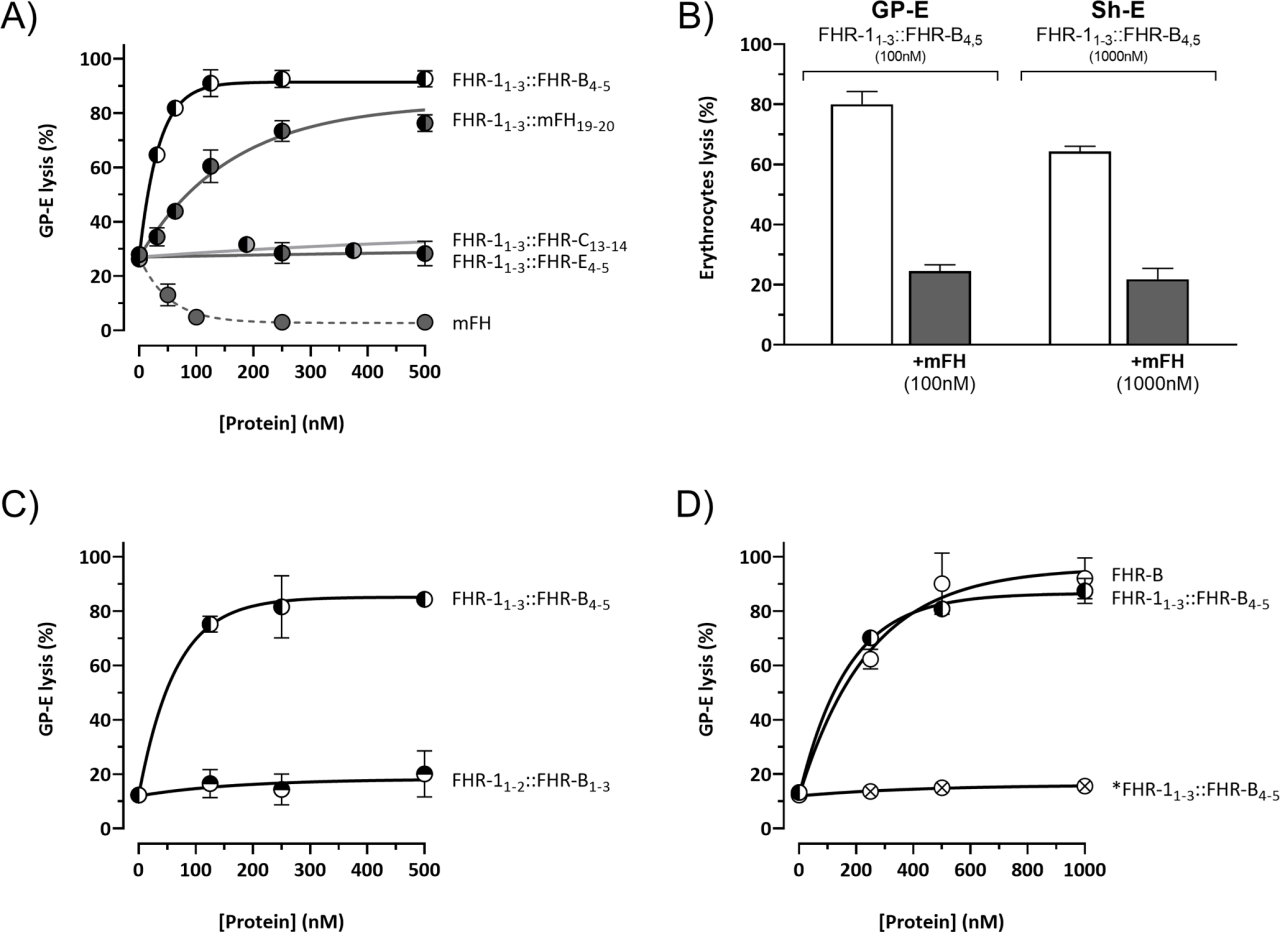
The C-terminal domain of FHR-B is functionally equivalent to the C-terminal domain of human FHR-1. **(A)** Hemolytic assay using GP-E and human serum of the hybrid proteins containing the N-terminal region of FHR-1 and the C-terminals domains of mFH (FHR-1_1-3_::mFH_19-20_), FHR-B (FHR-1_1-3_::FHR-B_4-5_), FHR-C (FHR-1_1-3_::FHR-C_14-15_) or FHR-E (FHR-1_1-3_::FHR-E_4-5_). Only hybrid proteins containing the C-terminal region of mFH and FHR-B exhibit de-regulation activity, measured as GP-E lysis, in these assays. **(B)** Hemolytic assay using GP-E or Sh-E and human serum to illustrate that the C-terminal region of FHR-B antagonizes FH regulation in both GP-E and Sh-E hemolysis assays. Figure also shows that addition of mFH antagonizes the de-regulation activity of FHR-B. **(C)** Hemolytic assay using GP-E and human serum to illustrate that the N-terminal domain of FHR-B (FHR-1_1-2_::FHR-B_1-3_) lacks FH de-regulation activity, measured as GP-E lysis. **(D)** Hemolytic assay using GP-E and human serum shows that full-length FHR-B exhibit similar FH de-regulation activity than the hybrid protein FHR-1_1-3_::FHR-B_4-5_. Since a monomeric variant of the hybrid protein FHR-1_1-3_::FHR-B_4-5_ (*FHR-1_1- 3_::FHR-B_4-5_) lacks FH de-regulation activity, these data suggests that FHR-B is a dimer. These experiments were performed in triplicate, and the figures present a representative result from at least three independent assays.

All three mouse proteins, FHR-B, FHR-C, and FHR-E, exhibit a very high sequence similarity to mFH (**Figure 1B**). As an initial approach to identify a potential functional homolog to FHR-1 in mice, we tested whether the C-terminal region of these mouse FHRs could replace the function of the C-terminal region of FHR-1 in Sh-E and GP-E hemolysis assays. To this end, we generated a series of recombinant proteins where the two C-terminal SCRs of FHR-B, FHR-C, and FHR-E proteins were fused to the three N-terminal SCRs of the FHR-1 protein. Additionally, we fused the two C-terminal SCRs of mouse FH to the three N-terminal SCRs of the FHR-1 protein as an additional reference (**Supplementary Figures 2A, B**). In GP-E hemolysis assays, using either human or mouse serum, we found that only the hybrid proteins with the C-terminal regions of FHR-B and mFH could compete with mouse FH for complement regulation (**Figure 3A**). Constructs with the C-terminal regions of FHR-C and FHR-E did not show any de-regulation activity in these assays. Consistent with earlier data (2), the construct with the C-terminus of FHR-B exhibited higher FH de-regulation activity than those with the C-terminus of mouse FH in both the Sh-E and GP-E hemolysis assays (**Figure 3B**). This contrasts with humans, where FHR-1 only shows de-regulation activity in the GP-E hemolysis assay, and this activity is significantly weaker than that of an FHR-1 with the C-terminal region of human FH (12). A construct combining the SCRs 1-2 of the N-terminal region of FHR-1 with the SCRs 1-3 of the N-terminal region of FHR-B (FHR-1_1-2_::FHR-B_1-3_) did not demonstrate complement de-regulation activity (**Figure 3C**).

We also compared the complement de-regulation activity of the hybrid protein FHR-1_1-3_::FHR-B_4-5_ with a recombinant form of the native FHR-B protein (rFHR-B). Both proteins demonstrated similar de-regulation activity in both hemolysis assays (**Figure 3D**, data now shown for Sh-E). Notably, the mutated *FHR-1_1-3_::FHR-B_4-5_, which is unable to form dimers through the FHR-1 N-terminal region, failed to show any de-regulation activity in these assays, strongly suggesting that the native FHR-B protein is a dimer.

### 3.3 N-terminal region of FHR-B contains a dimerization domain

**Figure 4:**
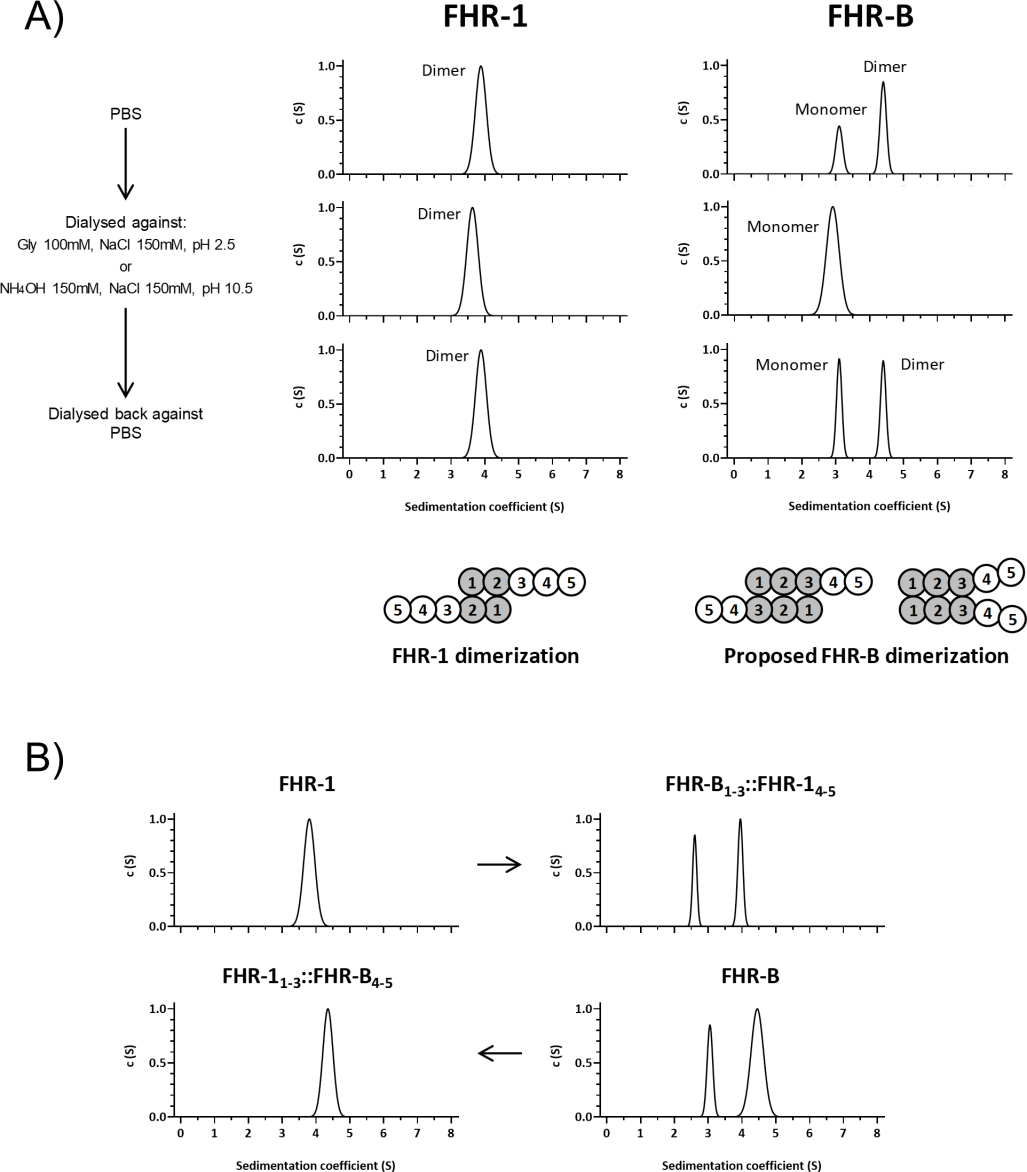
FHR-B dimerizes. **(A)** Full-length rFHR-B was analyzed by AUC, using FHR-1 for comparison. In PBS pH7, FHR-B resolves in 2 peaks, corresponding to FHR-B monomers and FHR-B dimers. FHR-B dimers disassembled at both high and low pH conditions, but reassemble when neutral pH is restored. Data also indicate that dimers of FHR-B have smaller sedimentation coefficient than dimers of FHR-1, which may suggest a more globular conformation of FHR-B. Diagrams illustrate the organization of the monomers in the FHR-1 dimers and a proposal for the organization of the FHR-B monomers in the dimers. **(B)** AUC experiments with FHR-1 and FHR-B hybrids in which we have switched the N-terminal domains. The data illustrate that the dimerization characteristics of FHR-1 and FHR-B are determined by their N-terminal domains.

We used AUC analyses to determine the size and multimeric organization of our rFHR-B, using FHR-1 and a monomeric FHR-1 mutant as reference markers. FHR-B consistently appeared as two molecular species: one with an experimental sedimentation coefficient of 3.1 S and a molecular weight of 38 kDa, and another with an experimental sedimentation coefficient of 4.6 S and a molecular weight of 69 kDa (**Figure 4A**). These values, corrected to standard conditions, correspond to the theoretical mass of an FHR-B monomer and an FHR-B dimer. The presence of these molecular species indicates that FHR-B dimerizes, and suggests a reversible interaction between FHR-B monomers.

To test this possibility, we exposed both FHR-1 and rFHR-B to acidic (pH 2.5) or basic (pH 10.5) conditions, analyzed them by AUC, dialyzed them back to physiological conditions, and re-analyzed them by AUC. Interestingly, while human FHR-1 dimers were unaffected by these treatments, mouse FHR-B dimers dissociated at low and high pH but re-associated under physiological conditions (**Figure 4A**). These data indicate a distinct dimerization mechanism for FHR-1 and FHR-B, consistent with the limited sequence similarity between the N-terminus of FHR-1 and FHR-B.

Our AUC analysis provided a friction ratio of approximately 1.3 for FHR-B, indicating that FHR-B is less elongated than FHR-1 (friction ratio of ~1.6), perhaps suggesting a different orientation of monomers in FHR-B dimers compared to FHR-1 (**Figure 4A**). Additional experiments showed that the hybrid protein FHR-B_1-3_::FHR-1_4-5_ is partially dimerized, while the mutated *FHR-1_1-3_::FHR-B_4-5_ does not form dimers (**Figure 4B**). These findings support that FHR-B circulates partially dimerized in mouse plasma, with the dimerization domain contained in the N-terminal region of the FHR-B molecule.

### 3.4 C-terminus of FHR-B mediates strong binding to native human and mouse C3 and to C3 activated fragments

**Figure 5:**
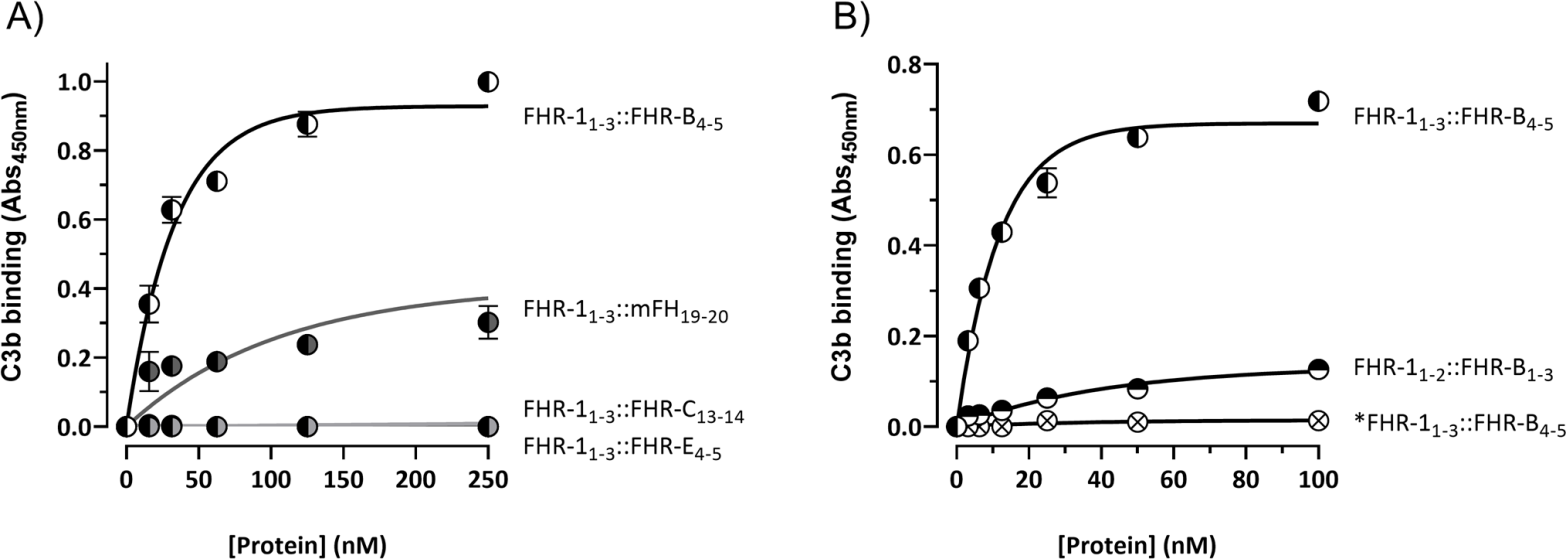
C-terminal domain of FHR-B binds strongly to C3b. **(A)** ELISA experiments performed with C3b-coated plates to analyze the capacity of the hybrid proteins including the C-terminal regions of FHR-B (FHR-1_1-3_::FHR-B_4-5_), FHR-C (FHR-1_1-3_::FHR-C_14-15_) or FHR-E (FHR-1_1-3_::FHR-E_4-5_) to bind C3b. A hybrid protein with the C-terminal region of mFH (FHR-1_1-3_::mFH_19-20_) was included as a reference. **(B)** Similar ELISA experiments as in A to show that an hybrid protein with the N-terminal domains of both FHR-1 and FHR-B (FHR-1_1-2_::FHR-B_1-3_) lacks C3b binding capacity. Similarly, these experiments show that the monomeric variant of the FHR-1_1-3_::FHR-B_4-5_ hybrid, called *FHR-1_1-3_::FHR-B_4-5_, lacks C3b binding capacity, which indicates that the strong FHR-B binding to C3b-coated surfaces requires FHR-B dimerization. These experiments were performed in triplicate, and the figures present a representative result from at least three independent assays.

Recent studies in our laboratory have shown that the C-terminus of FHR-1 includes a binding site that recognizes a region located in the TED domain of C3 (roughly equivalent to C3d) that is accessible for binding in native C3 (nC3) and in the C3 activated fragments C3b, iC3b and C3dg (12). To test whether the mouse FHR-B, FHR-C and FHR-E recognize also this binding site in the TED domain of C3 we first tested the interaction of our FHRs hybrid proteins with C3b immobilized to ELISA plates. These experiments showed that the FHR-1 hybrids with the C-terminal regions of FHR-B and mFH are the only proteins binding to C3b (**Figure 5A**), and that the construct with the C-terminal region of FHR-B binds to C3b much stronger than those with the C-terminal region of mFH (**Figure 5A**) or FHR-1 (**Supplementary Figure 3**). As expected, our experiments showed that the C-terminus of FHR-B binds also to nC3 and that a monomeric hybrid protein with the C-terminus of FHR-B, as well as a hybrid with the N-terminus of FHR-B failed to show significant binding to nC3 (**Supplementary Figure 4**) and C3b in these ELISAs (**Figure 5B**).

As a whole these data depict a FHR-B very similar to human FHR-1, with a C-terminal region involving surface ligands recognition that can replace the C-terminal region of FHR-1 and a N-terminal domain that mediates FHR-B dimerization.

### 3.5 FHR-B has much stronger de-regulation activity than human FHR-1

**Figure 6:**
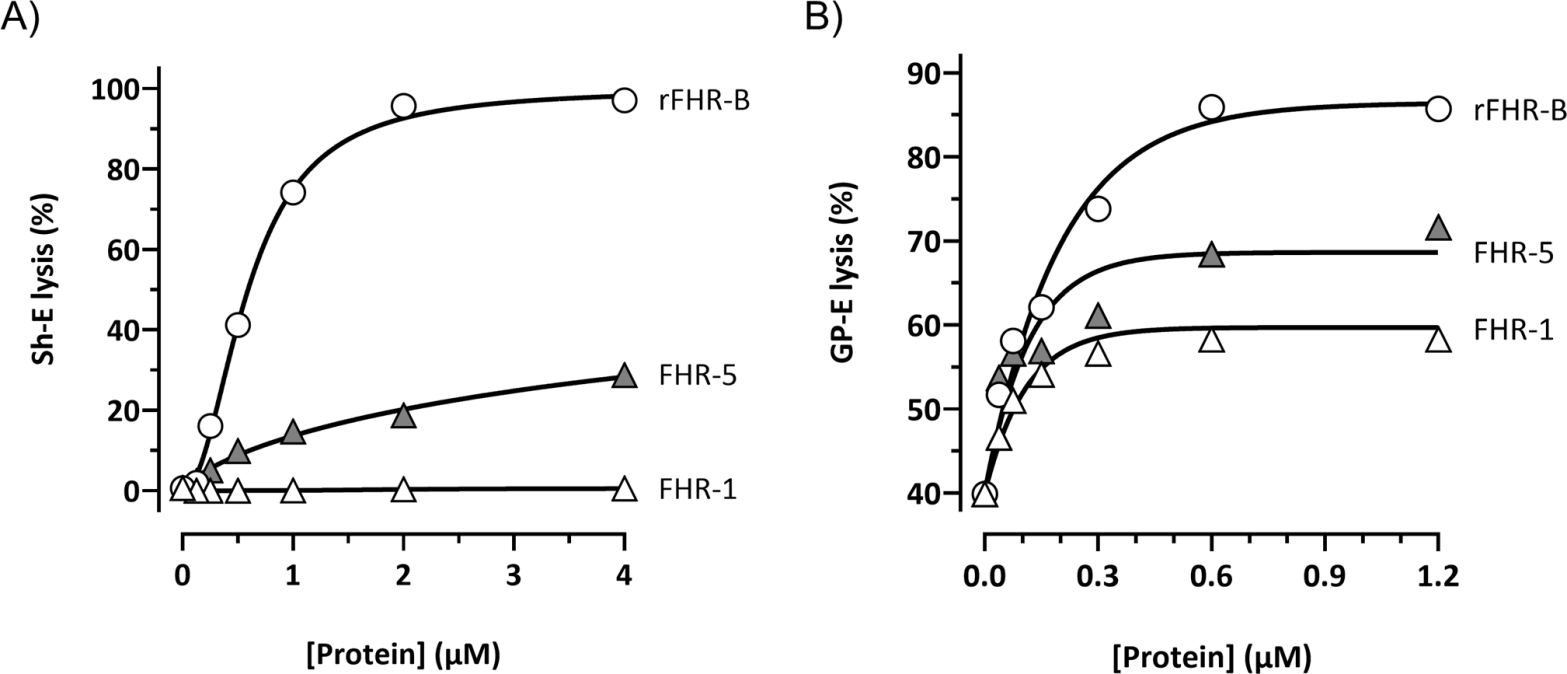
FHR-B promotes much stronger complement de-regulation in Sh-E and GP-E hemolysis assays than FHR-1 and FHR-5. **(A)** Hemolytic assay using Sh-E and mouse serum of the FHR-B, FHR-5 and FHR-1 proteins shows a higher capacity of FHR-B to promote complement de-regulation than FHR human proteins. De-regulating activity is measured as Sh-E lysis. **(B)** Hemolytic assay using GP-E and mouse serum of the FHR-B, FHR-5 and FHR-1 proteins shows a higher capacity of FHR-B to promote complement de-regulation than FHR human proteins. De-regulating activity is measured as GP-E lysis. These experiments were performed in duplicate, and the figures present a representative result from at least three independent assays.

**Figure 7:**
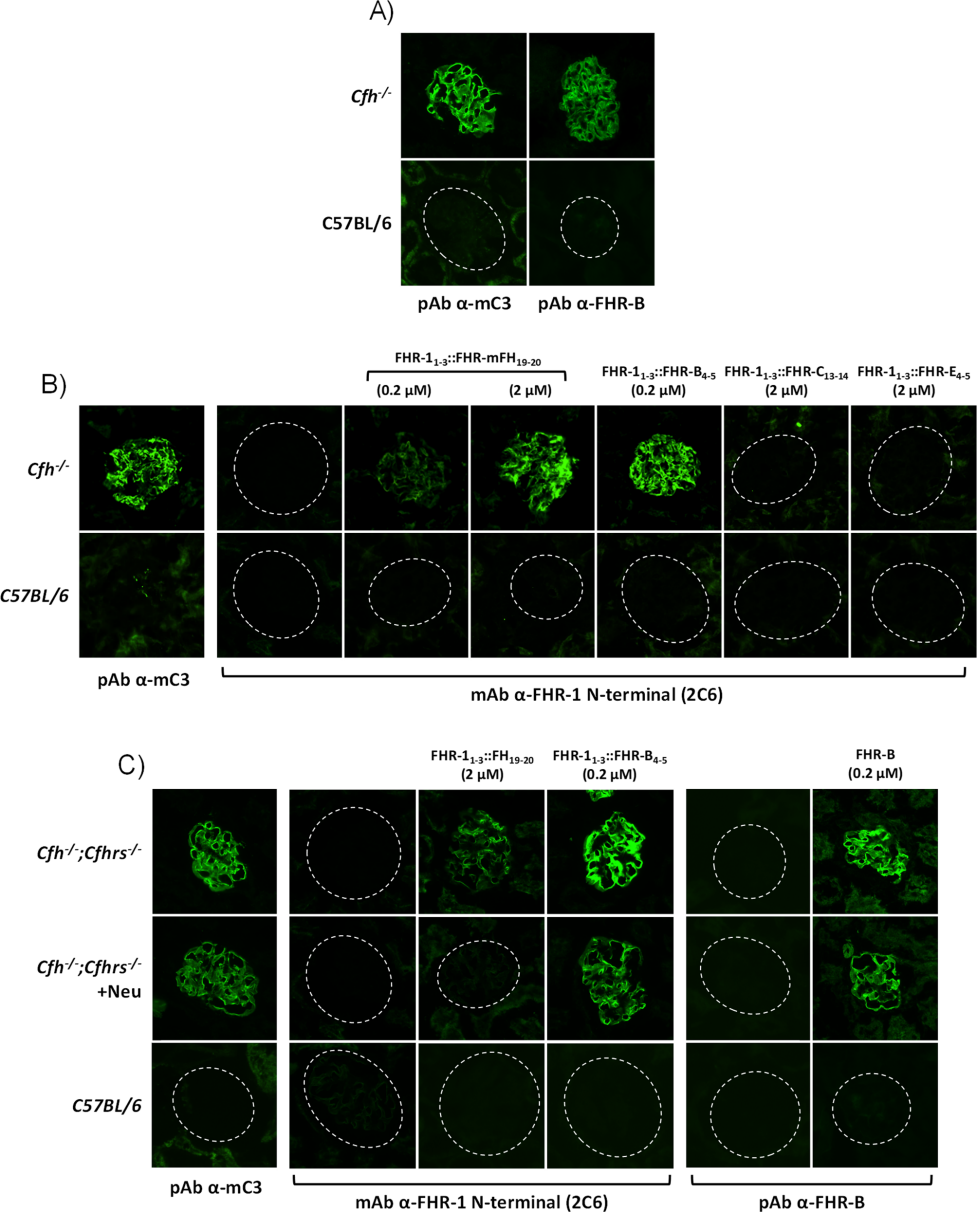
FHR-B binds strongly to C3-opsonized surfaces independently of the presence of sialic acids. **(A)** Cryostat kidney sections from C57BL/6 and *Cfh^-/-^* mice immunostained for mC3 and FHR-B reveal that FHR-B is naturally deposited in the C3-opsonized mice glomeruli. **(B)** Hybrid proteins with the C-terminal region of FHR-B (FHR-1_1-3_::FHR-B_4-5_), FHR-C (FHR-1_1-3_::FHR-C_14-15_), FHR-E (FHR-1_1-3_::FHR-E_4-5_) and mFH (FHR-1_1-3_::mFH_19-20_) were added to cryostat kidney sections of *Cfh^-/-^* mice and detected with a mouse monoclonal antibody directed to the N-terminal region of FHR-1 (2C6) that is present in all these hybrid proteins. Data illustrate that the hybrid FHR-1_1-3_::FHR-B_4-5_ shows a much stronger binding to C3-opsonized surfaces than the hybrid including the C-terminal region of mFH (FHR-1_1-3_::mFH_19-20_). No binding was observed with FHR-1_1-3_::FHR-C_14-15_ and FHR-1_1-3_::FHR-E_4-5_ hybrids. **(C)** Same binding assay on cryostat kidney sections of *Cfh^-/-^;Cfhrs^-/-^* mice was performed with the hybrid proteins FHR-1_1-3_::mFH_19-20_, FHR-1_1-3_::FHR-B_4-5_ and the full length rFHR-B. Much stronger binding was again observed for FHR-1_1-3_::FHR-B_4-5_ than FHR-1_1-3_::mFH_19-20_ and this binding was similar to that obtained for the rFHR-B protein. Interestingly, binding of FHR-1_1-3_::FHR-B_4-5_ and rFHR-B, but not of FHR-1_1-3_::mFH_19-20_ was preserved after desialylation of the cryostat kidney sections using *Clostridium perfringens* neuraminidase. Importantly, different antibodies were used to detect the hybrid proteins (2C6 anti-FHR-1 N-terminus) and full length rFHR-B (*in-house* pAb ant-FHR-B). As cryostat kidney sections of *Cfh^-/-^;Cfhrs^-/-^* mice were used, the *in-house* pAb ant-FHR-B shows no backgorund staining.

A direct comparison of the de-regulation activities of FHR-B, FHR-1 and FHR-5, using either human (**Supplementary Figure 5**) or mouse serum (**Figures 6A, B**), shows that FHR-B presents a much stronger de-regulation activity than FHR-1 or FHR-5. To investigate whether this is just a consequence of a higher binding to C3 activated fragments by the C-terminal region of FHR-B or it is implicated the binding to additional surface ligands, like sialic acids, we have used cryostat sections from *Cfh^-/-^* mice as described before (12). Glomeruli of these mice are C3-opsonized and provide, in addition to the C3b, iC3b and C3dg ligands, sialic acids and other ligands that may be relevant for a physiological interaction with FHR-B. Firstly, we showed that glomeruli for *Cfh^-/-^* mice stain for FHR-B whilst glomeruli from C57BL/6 mice do not, illustrating that endogenous FHR-B bind to C3 opsonized surfaces *in vivo* (**Figure 7A**). Using these *Cfh^-/-^* mice glomeruli we replicated the results of the hemolysis and C3b-binding assays with the FHR-1 hybrids, including the C-terminal regions of mFH, FHR-B, FHR-C and FHR-E, in the sense that the C-terminal of FHR-B binds to these glomeruli much better than the C-terminus of mFH and that the C-terminal of FHR-C and FHR-E do not bind to these glomeruli at all (**Figure 7B**). Removal of the sialic acids from the cell surfaces of the *Cfh^-/-^*; *Cfhrs^-/-^* mice glomeruli using *Clostridium perfringens* neuraminidase does not affect binding of the FHR-1_1-3_::FHR-B_4-5_ hybrid, but reduces significantly the binding of the hybrid with the C-terminus of mFH to these glomeruli (**Figure 7C**). These findings indicate that sialic acids do not contribute significantly to C-terminus of FHR-B binding to C3-opsonized mouse glomeruli, whereas, as previously described, they are critical for the binding of the C-terminus of mFH to these opsonized surfaces.

### 3.6 Surface-bound FHR-B protein promotes complement activation

**Figure 8:**
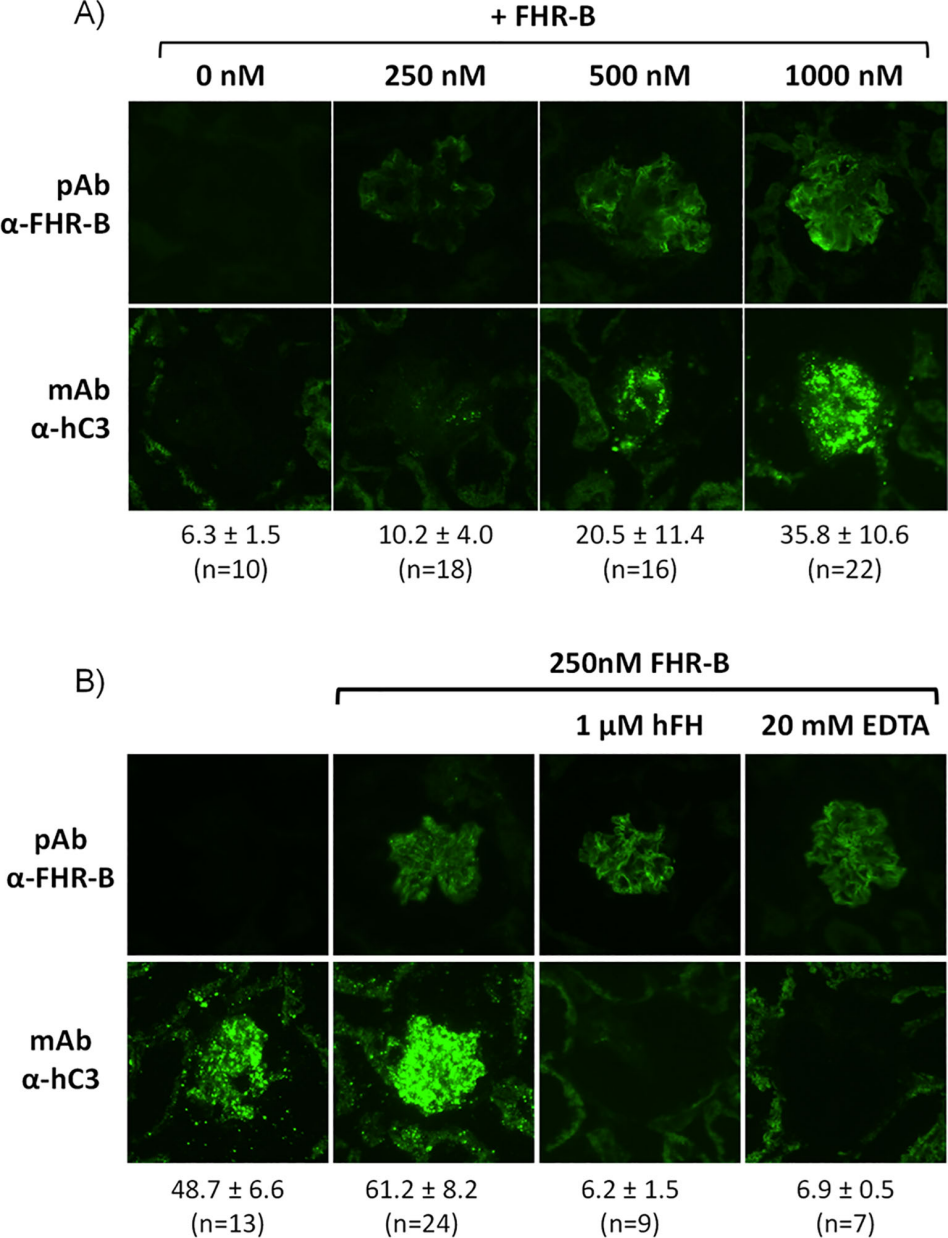
Surface bound FHR-B promotes complement activation through the alternative pathway. **(A)** Cryostat kidney sections from *Cfh^-/-^;Cfhrs^-/-^* mice were incubated with 10% NHSΔFHRs supplemented with 1.67 µM hFH (to prevent basal hC3 activation) in the absence and presence of varying amounts of rFHR-B. Increasing amounts of FHR-B led to increased FHR-B binding to the glomeruli, which in turn enhanced complement activation and hC3 deposition in the glomeruli. **(B)** Complement activation on cryostat kidney sections from *Cfh^-/-^;Cfhrs^-/-^* mice using no-hFH-supplemented NHSΔFHRs. Basal hC3 deposition is increased by surface-bound FHR-B. hC3 deposition promoted by the surface-bound FHR-B is inhibited by adding hFH or EDTA, indicating that C3 deposition requires formation of the alternative pathway C3-convertase and it is modulated by the FHR-B/FH ratio. The mean fluorescence intensity of C3 deposits is indicated below each panel in arbitrary units, together with the numbers of glomeruli analyzed.

To answer the question of whether the surface-bound FHR-B also promote complement activation and further increase C3 deposition like FHR-1, we used cryostat sections of *Cfh^-/-^*; *Cfhr^-/-^* mice as reported before (12). Kidney glomeruli in these mice are intensely opsonized and devoid of endogenous FHR-B (**Figure 8A**). Incubation of these glomeruli with either FHR-1_1-3_::FHR-B_4-5_ or rFHR-B results in strong binding of both molecules (**Figure 7C**) that upon incubation with NHSΔFHRs supplemented with 1.67 µM hFH (see Materials and Methods) promote significantly activation and deposition of human C3 (**Figure 8A**). The complement activation and deposition of C3 in these assays can be prevented by the addition of 20mM EDTA or by increasing the FH concentration, indicating that C3 deposition requires formation of the alternative pathway C3-convertase and it is modulated by the FHR-B/FH ratio (**Figure 8B**). Importantly, as we were testing the effect of hFH, these experiments were performed with no-hFH-supplemented NHSΔFHRs.

### 3.7 FHR-B competes binding of mFH to surface bound human C3b

**Figure 9:**
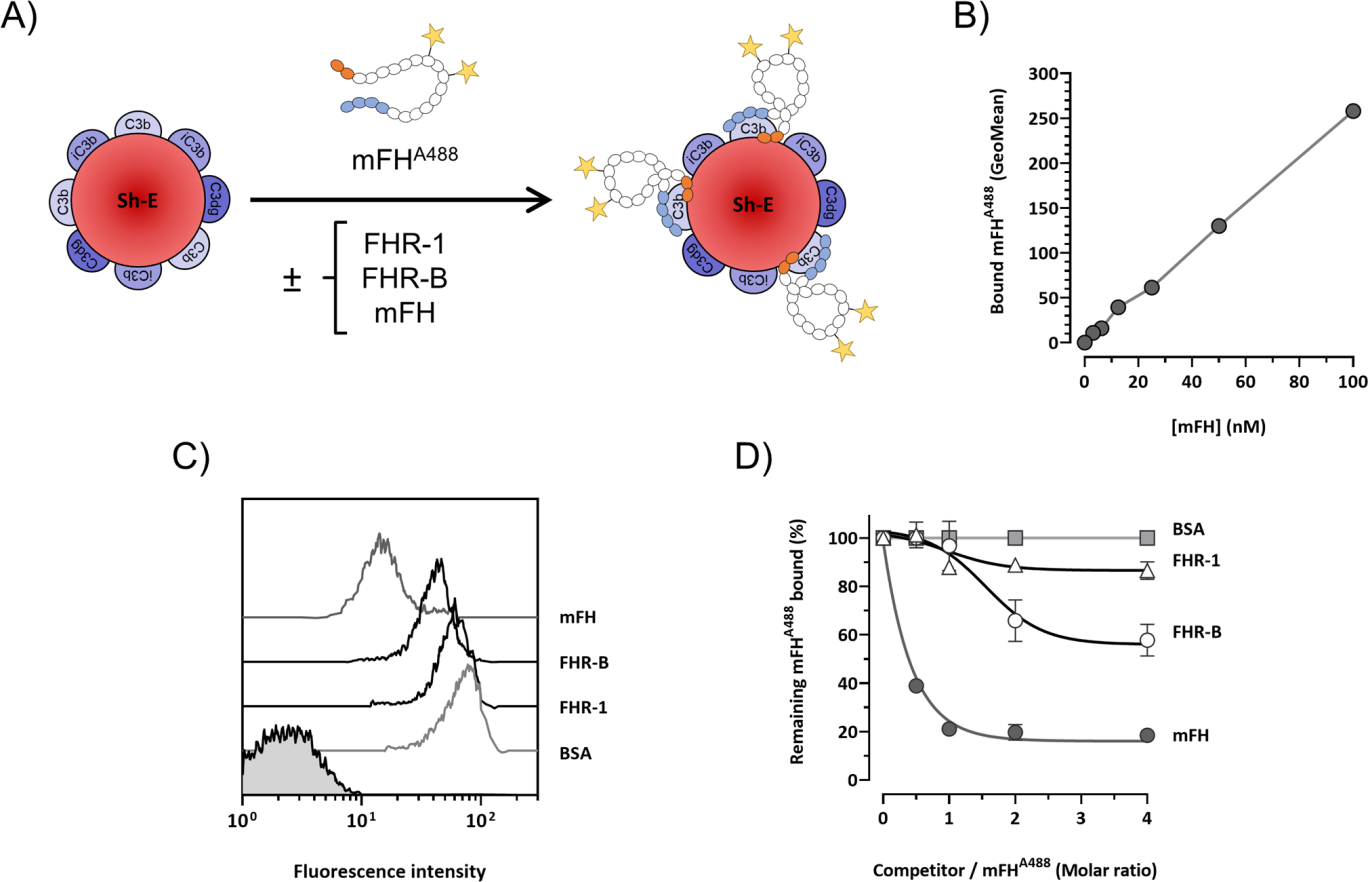
FHR-B competes binding of mFH to human C3b-opsonized surfaces. **(A)** Experiment design: mFH labeled with alexa F488 (mFH^AF488^) binds Sh-E coated with human C3 activated fragments in a doses-dependent manner and can be detected by flow cytometry. The capacity of rFHR-B to compete mFH binding was tested by incubating mFH^AF488^ in the presence of FHR-B. FHR-1 and non-labeled mFH were included as a negative and a positive control, respectively. **(B)** Calibration curve of the mFH^AF488^ binding to human C3b. **(C)** Competition of mFH^AF488^ binding to C3 coated Sh-E in the presence of mFH, rFHR-B, FHR-1 and BSA at a molar ratio of 1:4 (mFH^AF488^/competitor protein) measured as mean fluorescence intensity. **(D)** Competition of mFH^AF488^ binding to C3 coated Sh-E in the presence of mFH, rFHR-B, FHR-1 and BSA at different molar ratios. These experiments were performed in duplicate, and the figures present a representative result from at least three independent assays.

It has been reported earlier that FHR-B competes mFH for binding to surface-bound C3b (2), but this conclusion was based in experiments performed with C3b coated ELISA plates in the absence of the sialic acids that are critical for recognition of surface-bound C3b by mFH. We have recently developed a novel assay with opsonized Sh-E (C3-ShE) and used it to provide a formal demonstration that the FHR-1 protein does not compete with FH for C3b binding at physiological (sialic acid-containing) surfaces (13). Here, Sh-E were opsonized with human C3b and the binding of a mFH labelled with Alexa Fluor 488 (mFH^AF488^) to C3-ShE was challenged by the addition of increasing amounts of either mFH or FHR-B (**Figures 9A, B**). As negative controls, we used BSA and FHR-1. Our data show that, in contrast to FHR-1, mouse FHR-B competes binding of mFH to surface-bound human C3b (**Figures 9C, D**).

### 3.8 FHR-B enhances the cleavage of human C3b by FI in the presence of FH

**Figure 10:**
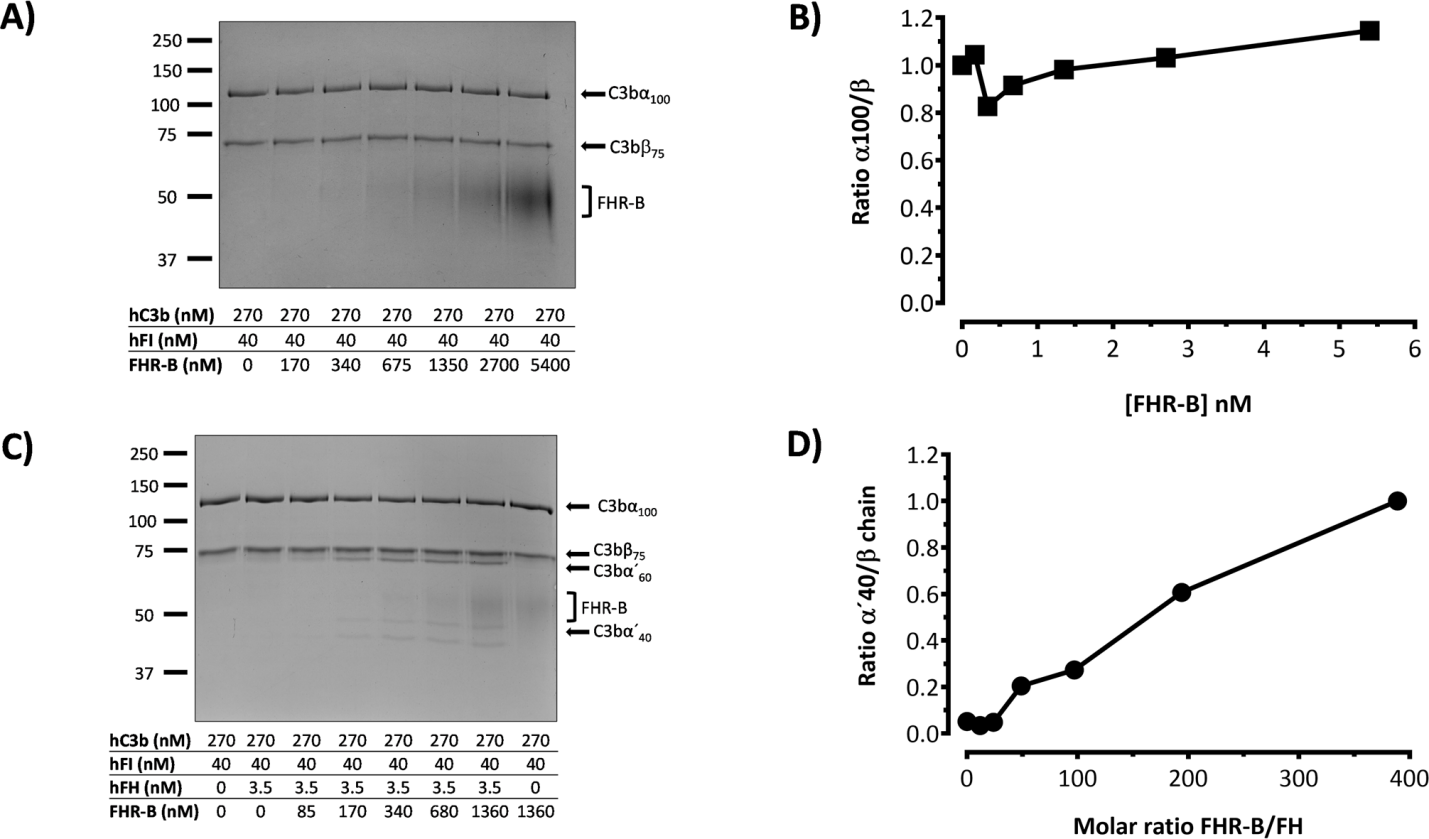
FHR-B does not have FI-cofactor activity for the cleavage of C3b, but enhances the FI-cofactor activity of limited amounts of FH. **(A)** Fluid phase assay with purified proteins shows that FHR-B does not have cofactor activity of FI in the proteolytic cleavage of C3b. hC3b 270nM and hFI 40nM were incubated with different concentrations of rFHR-B for 45 minutes at 37°C and loaded in an SDS-PAGE stained with Coomassie. No cleavage of the α’-chain of C3b was observed at any concentration. **(B)** Densitometry of the α’-chain and β-chains of C3b are represented as a α’-chain/β-chain ratio. **(C)** Fluid phase assay with purified proteins shows that FHR-B enhances the FI-cofactor activity of FH for the proteolytic cleavage of C3b in a dose-dependent manner. hC3b 270nM, hFI 40nM and hFH 3.5nM were incubated with different concentrations of rFHR-B for 45 minutes at 37°C and loaded in an SDS-PAGE stained with Coomassie. The FI-cofactor activity of FH is illustrated by the disappearance of the α’-chain of C3b and the appearance of the α60 and α40 C3b fragments. **(D)** Densitometry of the α40 and β-chains of C3b are represented in as a α40/β-chain ratio. The figures present a representative result from at least three independent assays.

Long time ago it was observed that FHR-3 and FHR-4 lacks FI-cofactor activity for the cleavage of C3b α-chain but can potentiate in a dose-dependent manner the FI-cofactor activity of FH (4). To confirm that FHR-B also lacks FI-cofactor activity for the cleavage of C3b we incubated C3b with FI and increasing amounts of FHR-B at 37°C for 45min, but we could not detect any cleavage of the α’ chain of C3b (**Figures 10A, B**). Interestingly, like for FHR-3 and FHR-4, if this same experiment is performed in the presence of very low concentrations of FH, the presence of FHR-B seems to potentiate in a dose-dependent manner the FI-cofactor activity of FH for the cleavage of the α’ chain of C3b (**Figures 10C, D**). This is a weak effect but may be physiological relevant at opsonized surfaces to prevent excessive generation of C3b.

### 3.9 Characterization of the FHR-B dimerization domain and its relevance to the FHR-B functionalities

**Figure 11:**
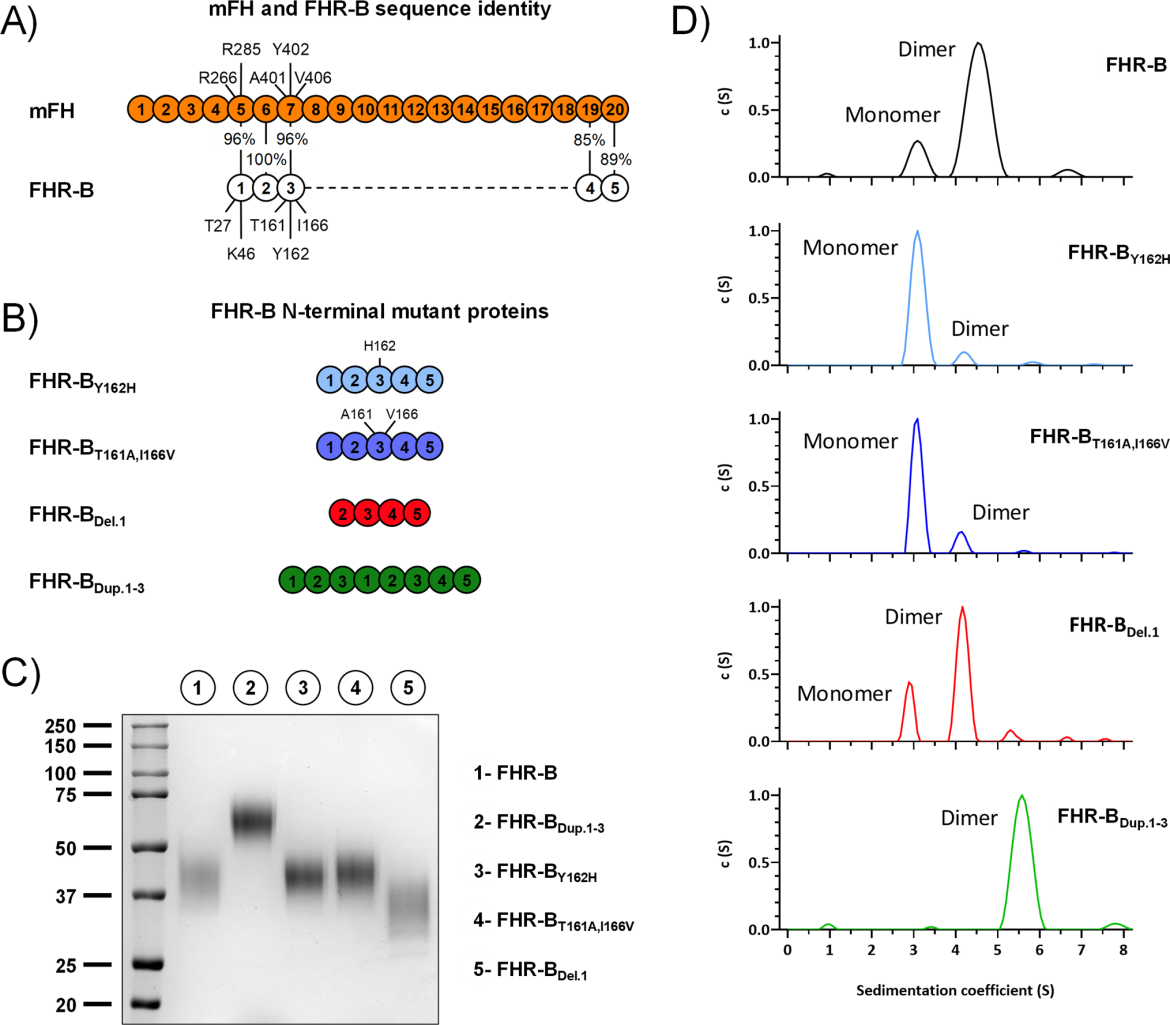
AUC analyses of FHR-B dimerization domain mutants. **(A)** Alignment between mFH and FHR-B, highlighting the differences in the three N-terminal region SCRs of FHR-B. **(B)** List of FHR-B mutants with alteration in the N-terminal region. **(C)** Coomassie stained SDS-PAGE under non-reducing conditions of the FHR-B N-terminal mutants produced. **(D)** AUC analyses (in PBS pH 7) of the FHR-B mutant proteins. FHR-B_T161A,I166V_, mimicking mFH SCR5-7 preserves an active dimerization domain, although the proportion of FHR-B dimers is reduced compared to the FHR-B native protein. Similar situation is observed when residue Tyr162 is mutated to His (FHR-B_Y162H_). As expected, a duplication of SCR1-3 of FHR-B (FHR-B_Dup.1-3_) results in a complete dimeric molecule. Notably, however, deletion of SCR1(FHR-B_Del.1_) has no impact in the dimerization capacity of FHR-B. As a whole, these data localize the FHR-B dimerization domain to SCR2 and SCR3.

The strongest sequence similarities of FHR-B SCRs 1-3 are with mouse FH SCRs 5-7 (with only four amino acid differences; **Figure 11A**), human FHR-3 SCRs 1-3 and human FH SCRs 5-7 (**Supplementary Figure 6**). Interestingly, two of the four differences (T161 and I166) between FHR-B and mFH are closely located in a region that also includes the Y162, a residue homologous to the disease-associated residue Y402 in human FH SCR7 (**Supplementary Figure 6**). Since we could not provide evidence of dimerization of mFH by AUC (data not shown) we postulated that the region including T161 and I166 was relevant for dimerization. To test this possibility, we substitute in the FHR-B protein the T161 and I166 residues for those in mouse FH. Additionally, we also tested if changing the Y162 to H162 impaired dimerization. The rational for this is that H402 in human FH is a strong risk factor for age-related macular degeneration and other human pathologies, but the functional consequences of changing Tyr for His at this position are still under debate. We also generated constructs of the FHR-B protein either lacking SCR1 or duplicating the N-terminal SCR1-3 region (**Figures 11B, C**). All these recombinant FHR-B mutants express normally and could be purify in sufficient quantities to be structurally and functionally characterized.

**Figure 12:**
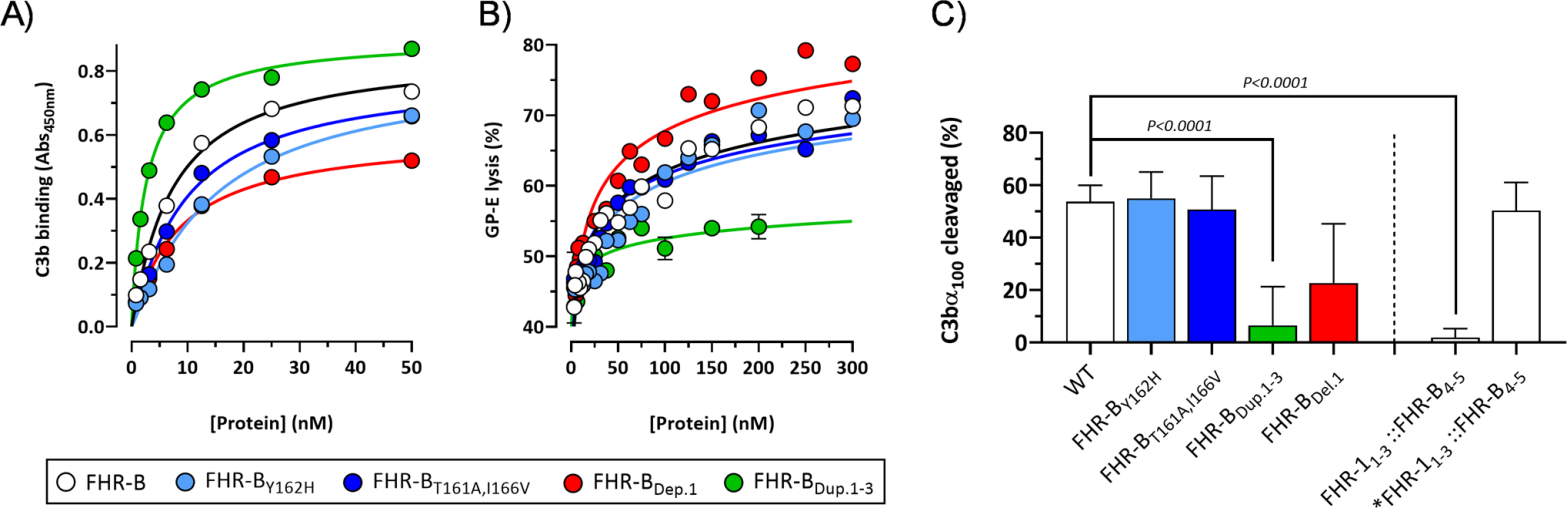
Functional analysis of FHR-B dimerization domain mutants. **(A)** ELISA experiments performed with human C3b-coated plates to analyze the capacity of the FHR-B mutant proteins including FHR-B_Y162H_, FHR-B_T161A,I166V_, FHR-B_Del.1_ and FHR-B_Dup.1-3_ to bind C3b. FHR-B protein was included as a reference. **(B)** A GP-E hemolysis assay with human serum was used to determine the FH de-regulation activity of the FHR-B N-terminal mutants. FHR-B_T161A,I166V_ and FHR-B_Y162H_ preserve this activity compared with the WT FHR-B protein. A slight increase in FH de-regulation activity was observed in the FHR-B mutant carrying a deletion of SCR1 (FHR-B_Del.1_), whereas these assays show that the FHR-B mutant with a duplication of the dimerization domain (FHR-B_Dup.1-3_) lacks almost completely FH de-regulation activity. **(C)** We also tested the capacity of the different FHR-B mutants to potentiate the FI-cofactor activity of limited amounts of FH. Data show that a fully dimerized FHR-B like FHR-B_Dup.1-3_ or FHR-1_1-3_::FHR-B_4-5_ lack this functionality, whereas a monomeric form of FHR-B or mutants with decreased proportion of dimers (FHR-B_T161A,I166V_ and FHR-B_Y162H_) preserve this activity. Data also suggests that FHR-B SCR1 is relevant for this activity. Student’s t-test statistical analysis for independent samples was performed using FHR-B as reference. P<0.05 was considered significant. These experiments were performed in triplicate, and the figures present a representative result from at least three independent assays.

AUC experiments show that both the mutant FHR B_T161A,I166V_ and the mutant FHR-B_Y162H_ present a reduce proportion of dimers, indicating that all these residues are involved in the dimerization process. In contrast, no differences in the proportion dimers/monomers were observed in the FHR-B mutant lacking SCR1. As expected, no monomers were observed in the FHR-B_Dup.SCR1-3_ mutant with a duplication of the dimerization domain (**Figure 11D**). Despite the experiments to determine the capacity of all mutants to bind C3b did not reveal significant differences that could not be explain by the increase/decrease of antigenic sites in the mutant FHR-B proteins (**Figure 12A**), the GP-E hemolytic assay to test competition of FH regulation by the FHR-B mutant proteins illustrated an unexpected complete lack of functional activity for the FHR-B_Dup.SCR1-3_ mutant (**Figure 12B**), which is in contrast with the data reported for the pathogenic variants of human FHR-1 and FHR-5 with duplicated dimerization domains (13, 21).

Interestingly, the FHR-1_1-3_::FHR-B_4-5_ hybrid does not potentiate the FI-cofactor activity of FH, whilst this activity is present in the mutant monomeric form *FHR-1_1-3_::FHR-B_4-5_ (**Figure 12C**). These data suggest that the capacity to enhance the cleavage of C3b by FI is exclusive of the FHR-B monomers, which are available in FHR-B, but not in FHR-1. Alternatively, the lack of this activity in the FHR-1_1-3_::FHR-B_4-5_ hybrid could be related to a different orientation of the monomers in the FHR-B and FHR-1 dimers (**Figure 4A**), with the later interfering this activity.

### 3.10 Plasma levels of FHR-B and antigenic similarities with other human and mouse proteins

**Figure 13:**
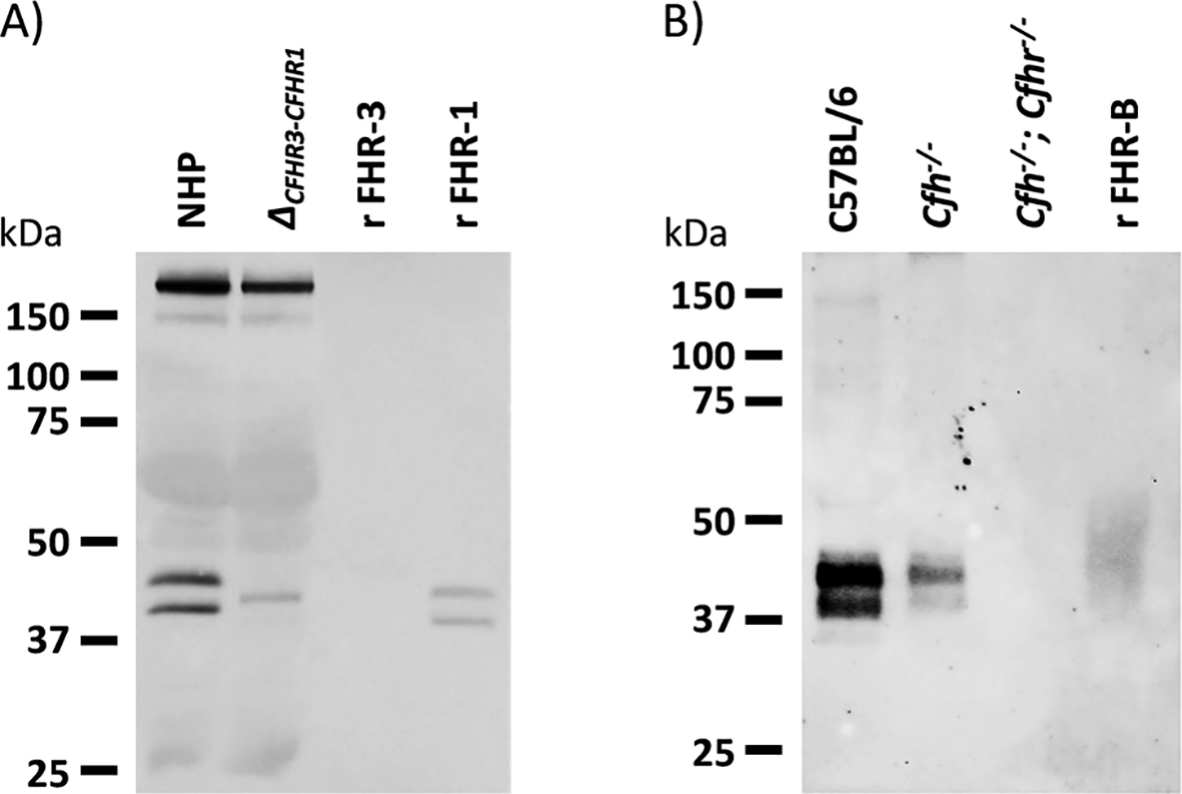
Western blot characterization of a rabbit polyclonal antibody raised against purified recombinant FHR-B. **(A)** Analysis of normal human plasma and a plasma from an individual deficient FHR-3 and FHR-1 show that the antibody cross-reacts with human FH, human FHL-1 and human FHR-1, but do not recognize human FHR-3. **(B)** The same antibody used in A was extensively adsorbed using a Sepharose column coupled to mFH and was tested in western blot with serum samples of C57BL/6, *Cfh^-/-^* and *Cfh^-/-^; Cfhr^-/-^* mice. No mouse FHR proteins other than FHR-B were detected. Notice the important decrease in FHR-B levels in the serum of *Cfh^-/-^* mice (see also **Figure 14**).

**Figure 14:**
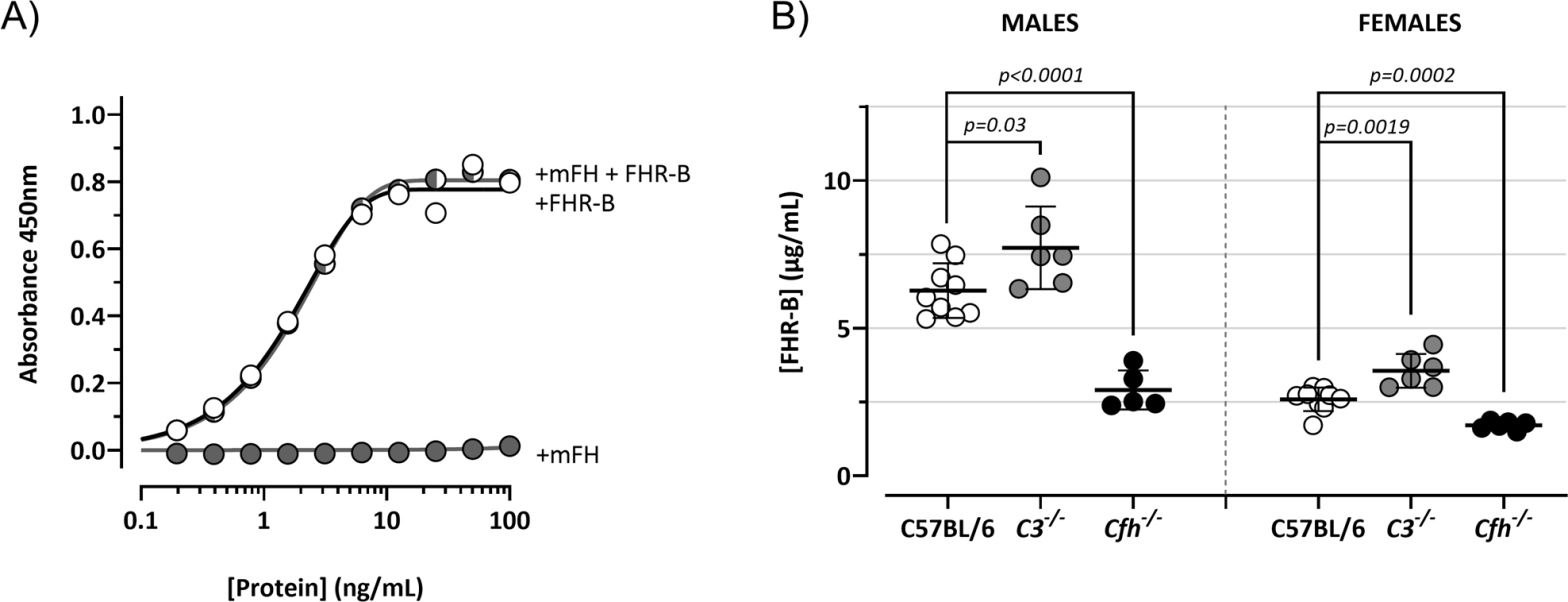
FHR-B levels in mouse plasma. **(A)** ELISA experiments to confirm that the rabbit polyclonal antibody generated *in-house* against the purified recombinant FHR-B protein and extensively adsorbed against mFH lacks mFH cross-reactivity. In these experiments *Cfh^-/-^; Cfhr^-/-^* mice plasma was reconstituted with different concentrations of mFH and/or FHR-B to show that while FHR-B was perfectly detected, mFH was not detected. **(B)** Using this ELISA we measure the plasma levels in male and female C57BL/6 mice. We also found that circulating levels of FHR-B were slightly increase in *C3^-/-^* mice and significantly decreased in *Cfh^-/-^* mice, suggesting that plasma FHR-B levels are influenced by the amount of C3 deposited in opsonized surfaces. Student’s t-test statistical analysis for independent samples was performed using C57BL6 mice serum as reference. P<0.05 was considered significant. These experiments were performed in duplicate, and the figures present a representative result from at least three independent assays.

We have tested the reactivity of our rabbit polyclonal antibody to FHR-B with human and mouse plasma proteins by western blot. Interestingly, we found that this antibody cross-reacts with human FH, FHL-1 and FHR-1 (**Figures 13A, B**). We also found a very weak reactivity with FHR-2 (not shown), but not with human FHR-3, FHR-4 and FHR-5. As expected, this antibody also recognizes mFH. An adsorbed version of this antibody in which we have removed the cross-reactivity with mFH (**Figure 14A**) was used to determine by ELISA the concentration of FHR-B in the plasma of C57Bl/6, *C3^-/-^* and *Cfh^-/-^* mice (**Figure 14B**). Levels of FHR-B in C57BL/6 males were approximately 7µg/mL (0.19µM), which is roughly 1:5 of the molar concentration of FH (1µM) in these animals. As for other complement proteins C57BL/6 females present much reduced levels of FHR-B in plasma than male mice. Interestingly, plasma levels of FHR-B are significantly increased in *C3^-/-^* mice and significantly decreased in *Cfh^-/-^* mice, which suggest that complement activation and surface deposition of C3 decreases the levels of circulating FHR-B (**Figure 14B**).

## 4 Discussion

Factor H-related proteins have been primarily studied in humans, and although our understanding of their role in the complement system remains incomplete, they are emerging as crucial modulators of complement activation at host and pathogen surfaces (12, 13, 22, 23). Dysregulation of the complement system mediated by the FHRs has also been implicated in several human diseases like aHUS (17, 18), C3G (8, 9), IgAN (24), AMD (12, 13, 25) and systemic lupus erythematosus (SLE) (26). In these conditions, it is postulated that FHRs mutations or copy number variations, altering their activity or plasma levels, disrupt the balance between complement activation (by the FHRs) and regulation (by FH), leading to excessive complement activity and subsequent tissue damage (12, 13). Generation of FHRs mice models will provide further insights into the mechanisms of the FHRs function and will facilitate the development of targeted therapeutic strategies for the associated pathologies. Our study was designed to enhance our understanding of the mouse FHRs and clear the path for the generation of such models. We confirmed the expression of FHR-B, FHR-C, and FHR-E proteins in mouse plasma. These three proteins present a high degree of sequence similarity with mFH in their C-terminal region (**Figure 1B**), but only the C-terminal region of FHR-B can replace the C-terminal region of FHR-1 in functional assays. Interestingly, FHR-B shows antigenic cross-reactivities and recapitulates most of the structural and functional characteristics of FHR-1, including its dimeric nature, although a potential different orientation of the monomers in the dimers emerges as a critical difference between FHR-B and FHR-1 (see below). Our data also illustrate significant similarities between FHR-B and FHR-5, particularly the capacity to compete binding to FH to surface-bound C3b (8). As a whole, we identify FHR-B as a promising candidate for modelling FHR-associated diseases in mice.

A main finding from these studies is to identify FHR-B as a dimeric protein. This characteristic and the high binding capacity of its C-terminal region for C3 and the C3 activated fragments confers FHR-B a very strong FH de-regulation activity. In fact, compared to FHR-1 and FHR-5, FHR-B presents much higher de-regulation activity than its human counterparts in both, the Sh-E and the GP-E hemolysis assays. This superior performance may be entirely related the high capacity of FHR-B to bind C3 and C3 activated fragments, but we cannot exclude yet a contribution of other surface ligands, like sialic acids, to this enhanced de-regulatory activity. Interestingly, and in contrast to the situation of human FHR-1 and FH, the C-terminal region of FHR-B binds much strongly to C3 opsonized surfaces than the C-terminal region of mFH with the important consequence that FHR-B competes efficiently the binding of mFH to C3b deposited in cell surfaces.

FHR-B is present in mouse plasma at concentration of 7μg/mL (0.19μM), which is roughly 5 times less molar concentration than mFH. This large difference probably eliminates the potential danger of FHR-B competing mFH and provoking non-specific and undesired complement activation at host cell surfaces, but the situation at opsonized surfaces may be different. Evidence suggests that on opsonized surfaces, the local concentration of FHR-B can be significantly elevated due to its strong binding affinity for C3b, iC3b, and C3dg. In fact, measurements of plasma levels of FHR-B in *C3^-/-^* and *Cfh^-/-^* animals provide evidence that plasma FHR-B levels are influenced by the degree of C3 deposition on surfaces, indicating that large amounts of FHR-B are likely confined to those surfaces (**Figure 14B**). Furthermore, these elevated concentrations of surface-bound FHR-B should also reduce the presence of FH by effectively competing the binding of FH to C3b on these surfaces. As a result, FHR-B can accumulate on C3-opsonized surfaces, potentially reaching concentrations that largely exceed those of FH. We, therefore, believe that while our experimental conditions involved much higher ratios of FHR-B to FH than typically found in plasma, these conditions may accurately represent the microenvironment on opsonized surfaces. In such contexts, the local concentration of FHR-B could be sufficiently high to enhance FH/FI-mediated cleavage of C3b. In whole, findings in this report support the notion that FHR-B enhances complement activation at opsonized surfaces, facilitating a continuous deposition of extra C3b on these surfaces. By enhancing the ability of mFH to inactivate C3b, FHR-B may also be redirecting the ensuing complement activation towards the production of iC3b and C3dg, which are the ligands for the complement receptors CR3 and CR4. As reported here, these FHR-B activities may favor opsonization over activation of the terminal pathway, which could be very relevant in all species, but especially in mice due to the known limited activity of the terminal pathway in this species.

The N-terminal region of FHR-B, which contains the dimerization domain, differs significantly from the N-terminal regions of human FHR-1, FHR-2, and FHR-5 in terms of amino acid sequence homology. This difference explains the distinct characteristics of the dimerization domain between FHR-B and the human proteins, such as its reversibility under acidic and basic conditions and the relative orientation of the monomers. This varying orientation may account for some of the functional differences between FHR-1 and FHR-B. For instance, the larger size of the FHR-1_1-3_::FHR-B_4-5_ dimer, with monomers in opposite orientation, may explain why native dimeric FHR-B enhances the cleavage of C3b by FI in the presence of FH, whereas the dimeric hybrid FHR-1_1-3_::FHR-B_4-5_ lacks this activity. Similarly, mouse FHR-B, which binds strongly to C3b, may require a weak dimerization domain to attract additional C3b molecules to a surface where monomeric FHR-B is already bound to C3b. In contrast, a completely dimerized FHR-B molecule with monomers in the same orientation, like the FHR-B_Dup.1-3_ mutant with a duplication of the dimerization domain, may be unable to generate a platform for forming additional C3-convertases with fluid phase C3 or C3b molecules, unlike FHR-1 with monomers in the opposite orientation.

Our experiments have identified several residues (T161, Y162, and I166) that contribute to, but are not indispensable for, the dimerization of FHR-B. This suggests that an extended surface in SCR3 is involved in FHR-B dimer formation. Notably, SCR3 in FHR-B is virtually identical to SCR7 in mouse FH and is well conserved in human FHR-3 SCR3 and human FH SCR7. This conservation implies that these other molecules may also present a weak dimerization domain in this region. The possibility that FH may dimerize under certain conditions has been previously suggested (27). It is tempting to speculate that substituting Tyr402 with His within SCR7 in human FH may impair this dimerization, potentially decreasing the regulatory activity of FH and its truncated isoform FHL-1, with significant pathological implications. Additionally, it is plausible that FHR-3 could form weak dimers, which might provide a mechanistic explanation for the association of an FHR-3::FHR-1 hybrid with C3G (28), making this molecule similar to the pathogenic FHR-1 mutant with a duplication of the dimerization domain (13).

In conclusion, our structural and functional characterization of mouse FHR-B has revealed several previously unknown features, particularly regarding the dimerization domain. This research suggests convergent evolution between mouse and human FHRs and underscores the critical role of dimerization in FHR functional activity. Importantly, the insights gained from the FHR-B dimerization domain could shed new light on the unresolved pathological associations of human FH and FHR variants, warranting further studies. Additionally, while our data identify FHR-B as a promising target for generating FHR disease models in mice, it also highlights notable differences between human FHR-1 and FHR-5 and mouse FHR-B that should be carefully considered before considering generating these mice models.

## Data Availability

The original contributions presented in the study are included in the article/**Supplementary Material**. Further inquiries can be directed to the corresponding author.

## References

[1] Vik DP Muñoz-Cánoves P Kozono H Martin LG Tack BF Chaplin DD. Identification and sequence analysis of four complement factor H-related transcripts in mouse liver. J Biol Chem. (1990) 265:3193–201. doi: 10.1016/s0021-9258(19)39753-4 1689298

[2] Cserhalmi M Csincsi ádám I Mezei Z Kopp A Hebecker M Uzonyi B. The murine factor H-Related protein FHR-B promotes complement activation. Front Immunol. (2017) 8:1145/BIBTEX. doi: 10.3389/FIMMU.2017.01145/BIBTEX 28974948 PMC5610720

[3] Hellwage J Skerka C Zipfel PF. Biochemical and functional characterization of the factor-H-related protein 4 (FHR-4). Immunopharmacology. (1997) 38:149–57. doi: 10.1016/S0162-3109(97)00075-1 9476126

[4] Hellwage J Jokiranta TS Koistinen V Vaarala O Meri S Zipfel PF. Functional properties of complement factor H-related proteins FHR-3 and FHR-4: binding to the C3d region of C3b and differential regulation by heparin. FEBS Lett. (1999) 462:345–52. doi: 10.1016/S0014-5793(99)01554-9 10622723

[5] Hellwage J Eberle F Babuke T Seeberger H Richter H Kunert A. Two factor H-related proteins from the mouse: Expression analysis and functional characterization. Immunogenetics. (2006) 58:883–93. doi: 10.1007/S00251-006-0153-Y/FIGURES/8 17028856

[6] Csincsi ÁI Szabó Z Bánlaki Z Uzonyi B Cserhalmi M Kárpáti É. FHR-1 binds to C-reactive protein and enhances rather than inhibits complement activation. J Immunol. (2017) 199:292–303. doi: 10.4049/jimmunol.1600483 28533443

[7] Csincsi ÁI Kopp A Zöldi M Bánlaki Z Uzonyi B Hebecker M. Factor H–related protein 5 interacts with pentraxin 3 and the extracellular matrix and modulates complement activation. J Immunol. (2015) 194:4963–73. doi: 10.4049/jimmunol.1403121 PMC4416742 25855355

[8] Goicoechea de Jorge E Caesar JJE Malik TH Patel M Colledge M Johnson S. Dimerization of complement factor H-related proteins modulates complement activation *in vivo* . Proc Natl Acad Sci. (2013) 110:4685–90. doi: 10.1073/pnas.1219260110 PMC3606973 23487775

[9] Tortajada A Yébenes H Abarrategui-Garrido C Anter J García-Fernández JM Martínez-Barricarte R. C3 glomerulopathy-associated CFHR1 mutation alters FHR oligomerization and complement regulation. J Clin Invest. (2013) 123:2434–46. doi: 10.1172/JCI68280DS1 PMC3668852 23728178

[10] Józsi M Tortajada A Uzonyi B Goicoechea de Jorge E Rodríguez de Córdoba S. Factor H-related proteins determine complement-activating surfaces. Trends Immunol. (2015) 36:374–84. doi: 10.1016/j.it.2015.04.008 25979655

[11] Antonioli AH White J Crawford F Renner B Marchbank KJ Hannan JP. Modulation of the alternative pathway of complement by murine factor H–related proteins. J Immunol. (2018) 200:316–26. doi: 10.4049/JIMMUNOL.1602017 PMC5736413 29187587

[12] Martin Merinero H Subías M Pereda A Gómez-Rubio E Juana Lopez L Fernandez C. Molecular bases for the association of FHR-1 with atypical hemolytic uremic syndrome and other diseases. Blood. (2021) 137:3484–94. doi: 10.1182/blood.2020010069 PMC8288665 33651882

[13] Márquez-Tirado B Gutiérrez-Tenorio J Tortajada A Lucientes Continente L Caravaca-Fontán F Malik TH. Factor H–related protein 1 drives disease susceptibility and prognosis in C3 glomerulopathy. J Am Soc Nephrol. (2022) 33:1137–53. doi: 10.1681/ASN.2021101318 PMC9161805 35545301

[14] Tortajada A Gutiérrez E Goicoechea de Jorge E Anter J Segarra A Espinosa M. Elevated factor H–related protein 1 and factor H pathogenic variants decrease complement regulation in IgA nephropathy. Kidney Int. (2017) 92:953–63. doi: 10.1016/j.kint.2017.03.041 28637589

[15] Cristobo I Larriba MJ de los Ríos V García F Muñoz A Casal JI. Proteomic analysis of 1α,25-Dihydroxyvitamin D3 action on human colon cancer cells reveals a link to splicing regulation. J Proteomics. (2011) 75:384–97. doi: 10.1016/J.JPROT.2011.08.003 21864731

[16] Käll L Canterbury JD Weston J Noble WS MacCoss MJ. Semi-supervised learning for peptide identification from shotgun proteomics datasets. Nat Methods. (2007) 4:923–5. doi: 10.1038/nmeth1113 17952086

[17] Valoti E Alberti M Tortajada A Garcia-Fernandez J Gastoldi S Besso L. A novel atypical hemolytic uremic syndrome-associated hybrid CFHR1/CFH gene encoding a fusion protein that antagonizes factor H-dependent complement regulation. J Am Soc Nephrol. (2015) 26:209–19. doi: 10.1681/ASN.2013121339 PMC4279739 24904082

[18] Goicoechea de Jorge E Tortajada A García SP Gastoldi S Merinero HM García-Fernández J. Factor H competitor generated by gene conversion events associates with atypical hemolytic uremic syndrome. J Am Soc Nephrol. (2018) 29:240–9. doi: 10.1681/ASN.2017050518 PMC5748918 28993505

[19] Val-Calvo J Luque-Ortega JR Crespo I Miguel-Arribas A Abia D Sánchez-Hevia DL. Novel regulatory mechanism of establishment genes of conjugative plasmids. Nucleic Acids Res. (2018) 46:11910–26. doi: 10.1093/NAR/GKY996 PMC6294495 30380104

[20] Schuck P. Size-distribution analysis of macromolecules by sedimentation velocity ultracentrifugation and lamm equation modeling. Biophys J. (2000) 78:1606–19. doi: 10.1016/S0006-3495(00)76713-0 PMC1300758 10692345

[21] Gale DP De Jorge EG Cook HT Martinez-Barricarte R Hadjisavvas A McLean AG. Identification of a mutation in complement factor H-related protein 5 in patients of Cypriot origin with glomerulonephritis. Lancet. (2010) 376:794–801. doi: 10.1016/S0140-6736(10)60670-8 20800271 PMC2935536

[22] Caesar JJE Lavender H Ward PN Exley RM Eaton J Chittock E. Competition between antagonistic complement factors for a single protein on N. meningitidis rules disease susceptibility. Elife. (2014) 3:e04008. doi: 10.7554/ELIFE.04008 25534642 PMC4273445

[23] Sándor N Schneider AE Matola AT Barbai VH Bencze D Hammad HH. The human factor H protein family – an update. Front Immunol. (2024) 15:1135490/BIBTEX. doi: 10.3389/FIMMU.2024.1135490/BIBTEX 38410512 PMC10894998

[24] Gharavi AG Kiryluk K Choi M Li Y Hou P Xie J. Genome-wide association study identifies susceptibility loci for IgA nephropathy. Nat Genet. (2011) 43:321–7. doi: 10.1038/ng.787 PMC3412515 21399633

[25] Hughes AE Orr N Esfandiary H Diaz-Torres M Goodship T Chakravarthy U. A common CFH haplotype, with deletion of CFHR1 and CFHR3, is associated with lower risk of age-related macular degeneration. Nat Genet. (2006) 38:1173–7. doi: 10.1038/ng1890 16998489

[26] Zhao J Wu H Khosravi M Cui H Qian X Kelly JA. Association of genetic variants in complement factor H and factor H-related genes with systemic lupus erythematosus susceptibility. PLoS Genet. (2011) 7:e1002079. doi: 10.1371/JOURNAL.PGEN.1002079 21637784 PMC3102741

[27] Perkins SJ Nan R Li K Khan S Miller A. Complement Factor H–ligand interactions: Self-association, multivalency and dissociation constants. Immunobiology. (2012) 217:281–97. doi: 10.1016/J.IMBIO.2011.10.003 22137027

[28] Malik TH Lavin PJ De Jorge EG Vernon KA Rose KL Patel MP. A hybrid CFHR3-1 gene causes familial C3 glomerulopathy. J Am Soc Nephrol. (2012) 23:1155–60. doi: 10.1681/ASN.2012020166/-/DCSUPPLEMENTAL PMC3380655 22626820

