# Supplementary material for: Functional and structural characterization of mouse Factor H-related B protein unveils a novel dimerization domain shared by FHR-B and FH
Source: Front Immunol. 2025 Jan 29;16:1522651. doi: 10.3389/fimmu.2025.1522651 (PMC11813886; doi:10.3389/fimmu.2025.1522651)
Supplement: Supplementary file 1 [file DataSheet1.docx]

**Supplementary material**

1. Supplementary Figure 1
2. Supplementary Figure 2
3. Supplementary Figure 3
4. Supplementary Figure 4
5. Supplementary Figure 5
6. Supplementary Figure 6
7. Supplementary Table 1
8. Supplementary Table 2

**Supplementary figure 1. The rabbit polyclonal antibody against purified mFH recognizes FH and another mouse plasma proteins, including mFHRs*.***

*Western Blot* of plasma-purified mFH and plasma of C57BL/6, FH KO (*Cfh* ^-/-^) and double FH and FHRs KO (*Cfh* ^-/-^*;Cfhrs* ^-/-^) mice using the rabbit polyclonal antibody anti-mFH developed *in-house*.

**Supplementary figure 2. Hybrid proteins generated for the study of mFHR proteins.**

A) schematic representation of the hybrid protein between FHR-1 and mFHRs.

B) Detection of the recombinant hybrid proteins in the culture supernatants by *Western Blot* using the mAb anti-hFHR-1 2C6.

**Supplementary figure 3. FHR-B C-terminal binds to C3b stronger than FHR-1.**

C3b-coated ELISA assay shows that the hybrid protein with the C-terminal domain of FHR-B (FHR-1_1-3_::FHR-B_4-5_) has a higher binding capacity to C3b than FHR-1. Points are mean ± SD of triplicates.

**Supplementary figure 4. FHR-B binds nC3 through the C-terminal region.**

ELISA plates coated with mouse nC3 were used to analyse the capacity of the hybrid proteins FHR-1_1-3_::FHR-B_4-5_ and FHR-1_1-2_::FHR-B_1-3_ to bind nC3. Only the protein with the C-terminal region of FHR-B binds to nC3. Points are mean ± SD of duplicates.

**Supplementary figure 5. FHR-B promotes complement de-regulation better than FHR-1 and FHR-5.**

A Sh-E hemolytic assay using normal human serum was performed to compare the capacity to promote complement de-regulation of FHR-B, FHR-1 and FHR-5. FHR-B shows the strongest capacity. As expected, FHR-1 does not promote lysis in this setting. Points are mean ± SD of duplicates.

**Supplementary figure 6.** **Amino acid alignment of hFH (SCR6-7), FHR-3 (SCR1,2), mFH (SCR 6,7) and FHR-B (SCR2,3).**

Differences between mFH and FHR-B, and between hFH and FHR-3 are highlighted in red. Residues corresponding to the human Y402H polymorphism and homologs are indicated in blue. “*” correspond to fully conserved residues; “:” conservation between groups of strongly similar properties; “.” conservation between groups of weakly similar properties; an empty space indicates non-conserved residues.

**Supplementary Table 1.**

**Top 20 proteins identified by nLC-MS/MS retained in the affinity column using the *in-house* pAb anti-mFH (*)**

| **Uniprot code** | **Description / Peptide sequence** | **Score** | **Coverage** | **Unique Peptides** | **Peptides** | **PSMs** |
| --- | --- | --- | --- | --- | --- | --- |
| P01027 | Complement C3 | 7645,19 | 44,56 | 79 | 79 | 298 |
| P11680 | Properdin | 6496,17 | 36,21 | 17 | 17 | 208 |
| P03987 | Ig gamma-3 chain C region | 5836,70 | 32,66 | 12 | 12 | 213 |
| E9Q8B5 | Complement factor H related protein C | 5741,34 | 40,96 | 25 | 26 | 165 |
|  | RVEYSHGEVVGYDcKPR |  |  |  |  | 4 |
|  | KIScPPPPQIPNTQVIETTVK |  |  |  |  | 9 |
|  | IScPPPPQIPNTQVIETTVK |  |  |  |  | 7 |
|  | LNDKLDYEcLIGYENEYK |  |  |  |  | 4 |
|  | VGPDSVQcYHFGWSPSFPTcK |  |  |  |  | 5 |
|  | TDcDILPTIENAIIR |  |  |  |  | 7 |
|  | LYYEEILRPNFPVSIGNK |  |  |  |  | 6 |
|  | NIYSHSGEDIEFEcK |  |  |  |  | 11 |
|  | ScERPVFENSVTK |  |  |  |  | 1 |
|  | IPcSQPPTIEHGSIK |  |  |  |  | 25 |
|  | cTVQGWEPEVPcVR |  |  |  |  | 13 |
|  | VGDLLEFScR |  |  |  |  | 13 |
|  | STDIEAIKPK |  |  |  |  | 2 |
|  | cGPPPPIDNGDITSLSLPEYEPFSSVDYQcQK |  |  |  |  | 2 |
|  | TcGDIPELEHGSVK |  |  |  |  | 16 |
|  | TcSVSDIEIENGFFSESFR |  |  |  |  | 2 |
|  | VEYSHGEVVGYDcKPR |  |  |  |  | 6 |
|  | cVATDQLEK |  |  |  |  | 3 |
| E9Q8B5; Q4LDF6 | IYVQGQSLK |  |  | NO |  | 2 |
|  | GSITcTYYGWSDTPScYEIEcSVPILDR |  |  |  |  | 2 |
|  | ScEFPQFK |  |  |  |  | 8 |
|  | GSITcTYYGWSDTPScYEIEcSVPILDRK |  |  |  |  | 1 |
|  | WNPEPNcIR |  |  |  |  | 3 |
|  | cISGTINYPIcE |  |  |  |  | 3 |
|  | WQSLPHcIEK |  |  |  |  | 7 |
|  | AGQVAScAQPPEIPNGEINGAK |  |  |  |  | 3 |
| P01029 | Complement C4-B | 4907,79 | 26,87 | 51 | 51 | 183 |
| P07724 | Serum albumin | 3918,02 | 45,39 | 31 | 31 | 128 |
| Q02105 | Complement C1q subcomponent subunit C | 3773,32 | 30,89 | 7 | 7 | 129 |
| Q61406 | Complement factor H related protein E | 3566,10 | 64,14 | 14 | 14 | 136 |
|  | cGPPPPIDNGDITSLSLPVYASLSSVEYQcQK |  |  |  |  | 4 |
|  | VYIQSGEDIEFGcKPR |  |  |  |  | 13 |
|  | ILYYScEYNFASPSNSFWTR |  |  |  |  | 3 |
|  | ITcTESGWSPTPK |  |  |  |  | 8 |
|  | HGILYDEK |  |  |  |  | 10 |
|  | cGPPPPIDNGDITSLSLLEYEPLSSVEYQcQNYYVLK |  |  |  |  | 3 |
|  | KNEPFSSVLSGK |  |  |  |  | 33 |
|  | WSEPPTcLSAcVISEAImER |  |  |  |  | 3 |
|  | WSEPPTcIYPTGK |  |  |  |  | 19 |
|  | cISTNPTGK |  |  |  |  | 5 |
|  | NEPFSSVLSGK |  |  |  |  | 20 |
|  | tQcINGHINYPTcmLNHNTFIH |  |  |  |  | 4 |
|  | AKGSLPFR |  |  |  |  | 1 |
|  | GEVSLcDFPK |  |  |  |  | 10 |
| P01864 | Ig gamma-2A chain C region secreted form | 2806,71 | 18,21 | 6 | 6 | 118 |
| P28665 | Murinoglobulin-1 | 2371,35 | 22,09 | 14 | 30 | 90 |
| Q00896 | Alpha-1-antitrypsin 1-3 | 2257,02 | 22,09 | 4 | 10 | 92 |
| P14106 | Complement C1q subcomponent subunit B | 2235,19 | 25,69 | 6 | 6 | 65 |
| Q4LDF6 | Complement factor H related protein B | 2206,52 | 62,05 | 13 | 14 | 101 |
|  | TcGPPPPIDNGDITSLSLPEYEPLSSVDYQcQK |  |  |  |  | 4 |
|  | SDDEIRYEcNYGFYPVTGSTVSK |  |  |  |  | 5 |
|  | LYYEESLRPNFPVSIGNK |  |  |  |  | 13 |
|  | cTLKPcEFPQFK |  |  |  |  | 5 |
|  | IYAHSGEDIEFEcKR |  |  |  |  | 11 |
|  | IYAHSGEDIEFEcK |  |  |  |  | 12 |
|  | cTAQGWEPEVPcVR |  |  |  |  | 18 |
|  | cDNGFSPPSGYSWDYLR |  |  |  |  | 5 |
|  | YEcNYGFYPVTGSTVSK |  |  |  |  | 10 |
|  | TcSPPYILNGIYTPHR |  |  |  |  | 1 |
| Q4LDF6; E9Q8B5 | IYVQGQSLK |  |  | NO |  | 2 |
|  | cTPTGWIPVPR |  |  |  |  | 12 |
|  | IIHKSDDEIRYEcNYGFYPVTGSTVSK |  |  |  |  | 1 |
|  | KcVFHYVENGDSTYWEK |  |  |  |  | 2 |
| P98086 | Complement C1q subcomponent subunit A | 1998,58 | 23,67 | 7 | 7 | 68 |
| Q00897 | Alpha-1-antitrypsin 1-4 | 1951,14 | 22,28 | 2 | 9 | 74 |
| P08607 | C4b-binding protein | 1824,03 | 34,12 | 14 | 14 | 72 |
| P97290 | Plasma protease C1 inhibitor | 1772,18 | 24,80 | 13 | 13 | 80 |
| P01837 | Immunoglobulin kappa constant | 1426,65 | 60,75 | 8 | 8 | 51 |
| P60710 | Actin, cytoplasmic 1 | 1378,12 | 30,67 | 10 | 10 | 43 |
| P20918 | Plasminogen | 1324,14 | 37,68 | 27 | 27 | 53 |

(*) Peptides unique to the mFHRs proteins are displayed below each one.

**Supplementary Table 2.**

**Top 20 proteins identified by nLC-MS/MS retained in the affinity column using a non-immune rabbit IgG ***

| **Uniprot code** | **Description / Peptide sequence** | **Score** | **Coverage** | **Unique Peptides** | **Peptides** | **PSMs** |
| --- | --- | --- | --- | --- | --- | --- |
| P07724 | Serum albumin | 6754,04 | 66,94 | 1 | 39 | 39 |
| P01872 | Ig mu chain C region | 4507,31 | 47,58 | 1 | 21 | 21 |
| P11276 | Fibronectin | 2944,67 | 29,27 | 1 | 53 | 53 |
| Q921I1 | Serotransferrin | 2341,82 | 51,65 | 1 | 39 | 39 |
| Q02105 | Complement C1q subcomponent subunit C | 2334,81 | 30,89 | 1 | 8 | 8 |
| P98086 | Complement C1q subcomponent subunit A | 2298,01 | 30,61 | 1 | 10 | 10 |
| Q61838 | Pregnancy zone protein | 2201,39 | 37,19 | 2 | 45 | 45 |
| P14106 | Complement C1q subcomponent subunit B | 2121,59 | 28,06 | 1 | 6 | 6 |
| Q91X72 | Hemopexin | 1386,52 | 42,17 | 1 | 18 | 18 |
| P01867 | Ig gamma-2B chain C region | 1134,90 | 32,18 | 1 | 9 | 9 |
| P03987 | Ig gamma-3 chain C region | 936,92 | 33,42 | 1 | 10 | 10 |
| P01843 | Ig lambda-1 chain C region | 859,14 | 61,90 | 1 | 4 | 4 |
| P32261 | Antithrombin-III | 820,62 | 41,94 | 1 | 20 | 20 |
| P01837 | Immunoglobulin kappa constant | 769,38 | 41,12 | 1 | 6 | 6 |
| Q9QWK4 | CD5 antigen-like | 762,75 | 41,19 | 1 | 12 | 12 |
| Q00897 | Alpha-1-antitrypsin 1-4 | 669,18 | 26,39 | 1 | 3 | 10 |
| O70362 | Phosphatidylinositol-glycan-specific phospholipase D | 607,61 | 20,67 | 1 | 16 | 16 |
| P22599 | Alpha-1-antitrypsin 1-2 | 586,59 | 25,42 | 1 | 3 | 10 |
| Q00896 | Alpha-1-antitrypsin 1-3 | 568,20 | 22,33 | 2 | 4 | 9 |
| Q06890 | Clusterin | 558,19 | 28,79 | 1 | 12 | 12 |

(*) No mouse FHRs proteins were retained by the control Sepharose column coupled to a non-immune rabbit IgG. Importantly, only 1-2 unique peptides were identified for these 20 top proteins, indicating they are just experimental noise.
